# Resolving the α-glycosidic linkage of arginine-rhamnosylated translation elongation factor P triggers generation of the first Arg^Rha^ specific antibody†
†Electronic supplementary information (ESI) available: Experimental details and chemical compound characterization. CCDC 1469830. For ESI and crystallographic data in CIF or other electronic format see DOI: 10.1039/c6sc02889f



**DOI:** 10.1039/c6sc02889f

**Published:** 2016-07-21

**Authors:** Xiang Li, Ralph Krafczyk, Jakub Macošek, Yu-Lei Li, Yan Zou, Bernd Simon, Xing Pan, Qiu-Ye Wu, Fang Yan, Shan Li, Janosch Hennig, Kirsten Jung, Jürgen Lassak, Hong-Gang Hu

**Affiliations:** a Department of Organic Chemistry , School of Pharmacy , Second Military Medical University , Shanghai 200433 , China . Email: huhonggang_fox@msn.com; b Department of Biology I, Microbiology , Ludwig Maximilians-Universität München , Munich , Germany; c Center for Integrated Protein Science Munich , Ludwig-Maximilians-Universität München , Munich , Germany . Email: juergen.lassak@lmu.de; d Structural and Computational Biology Unit , EMBL Heidelberg , Heidelberg 69117 , Germany; e School of Pharmacy , Wei Fang Medical University , Shandong 261053 , China; f Institute of Infection and Immunity , Taihe Hospital , Hubei University of Medicine , Shiyan , Hubei 442000 , China

## Abstract

A previously discovered posttranslational modification strategy – arginine rhamnosylation – is essential for elongation factor P (EF-P) dependent rescue of polyproline stalled ribosomes in clinically relevant species such as *Pseudomonas aeruginosa* and *Neisseria meningitidis*. However, almost nothing is known about this new type of *N*-linked glycosylation. In the present study we used NMR spectroscopy to show for the first time that the α anomer of rhamnose is attached to Arg32 of EF-P, demonstrating that the corresponding glycosyltransferase EarP inverts the sugar of its cognate substrate dTDP-β-l-rhamnose. Based on this finding we describe the synthesis of an α-rhamnosylated arginine containing peptide antigen in order to raise the first *anti*-rhamnosyl arginine specific antibody (*anti*-Arg^Rha^). Using ELISA and Western Blot analyses we demonstrated both its high affinity and specificity without any cross-reactivity to other *N*-glycosylated proteins. Having the *anti*-Arg^Rha^ at hand we were able to visualize endogenously produced rhamnosylated EF-P. Thus, we expect the antibody to be not only important to monitor EF-P rhamnosylation in diverse bacteria but also to identify further rhamnosyl arginine containing proteins. As EF-P rhamnosylation is essential for pathogenicity, our antibody might also be a powerful tool in drug discovery.

## Abstract 2

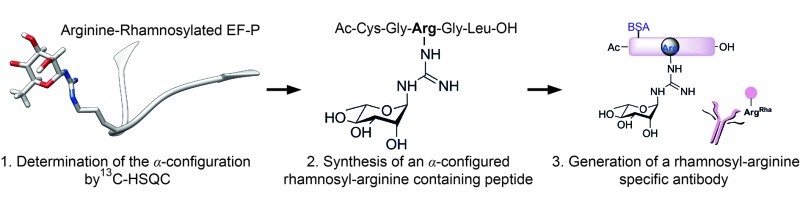
Here we describe a potent tool to investigate arginine rhamnosylation and develop novel antibiotics.

## Introduction

**Fig. 1:**
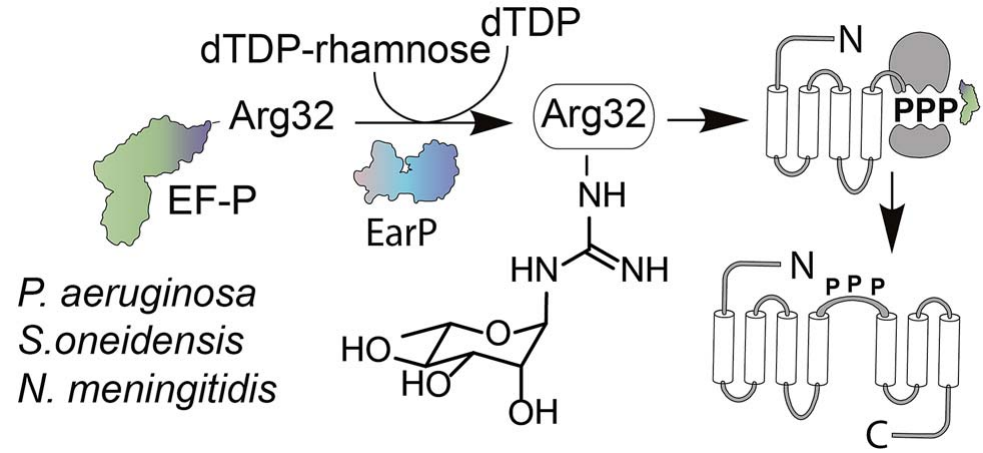
EF-P arginine rhamnosylation and mode of action. Certain bacteria including *P. aeruginosa*, *S. oneidensis*, and *N. meningitidis* encode an EF-P variant with an invariant arginine at position 32. The glycosyltransferase EarP activates EF-P by rhamnosylation of Arg32 using dTDP-β-l-rhamnose as substrate. EF-P and its rhamnose modification stimulate proline–proline peptide bond formation thereby alleviating ribosome stalling at polyproline stretches. EF-P = translation elongation factor P; EarP = EF-P specific arginine rhamnosyl transferase for posttranslational activation.

Glycosylation is one of the most important posttranslational modifications (PTMs) of proteins in biological systems^1,2^ and is associated with numerous biological processes including viral and bacterial infection, cancer metastasis, inflammatory response, innate and adaptive immunity, as well as many signaling pathways.^3,4^ For a long time, protein glycosylation was considered to be restricted to eukaryotes. Today it is well accepted that also bacteria including important human pathogens contain a large number of *O*- and *N*-linked glycoproteins.^5,6^ However until 2013 only one case of a sugar being added to arginine was reported.^7^ At that time, two research groups discovered independently that the type III secretion system effector NleB, of enteropathogenic *Escherichia coli* (EPEC) acts as arginine-*N*-acetylglucosamine (Arg^GlcNAc^) transferase on human death receptor domains, thereby interfering with the host defense.^8,9^ We elucidated that another type of arginine glycosylation plays an important role in the activation of the specialized translation elongation factor EF-P, which alleviates ribosome stalling at polyproline sequences (Fig. 1).^10–13^

For effective ribosome rescue certain bacteria, including not only the versatile γ-proteobacterium *Shewanella oneidensis* MR-1 but also the important human pathogens *Pseudomonas aeruginosa* and *Neisseria meningitidis*, post-translationally rhamnosylate a conserved Arg32.^14–16^ When EF-P is bound to the ribosome the rhamnosylated arginine protrudes towards the peptidyltransferase center thereby contributing to the favorable positioning of the peptidyl-Pro-tRNA and stabilization of the CCA-end of the prolyl-tRNA.^14,17,18^ Loss of the rhamnose modification abolishes the pathogenicity of *P. aeruginosa* ^14^ and increases its susceptibility to certain antibiotics.^15^ Thus inhibition of EF-P rhamnosylation might be a novel strategy to selectively suppress virulence. However, little is known about the corresponding glycosyltransferase EarP or arginine rhamnosylation itself. Here we used NMR spectroscopy and found the rhamnosyl moiety on the protein acceptor EF-P in the α-configuration, unambiguously demonstrating that EarP is an inverting glycosyltransferase. Based on this result, we report the generation of the first high-affinity *anti*-Arg^Rha^-specific antibody that allowed us to detect rhamnosylated EF-P even from crude cell lysates. With this molecular tool in hand, we will not only be able to improve our understanding of EarP mediated EF-P rhamnosylation, but the antibody might also help to develop new potent targeted antibiotics and to unveil other arginine rhamnosylated proteins.

## Results and discussion

### EarP mediates an inverting glycosyl transfer reaction

**Fig. 2:**
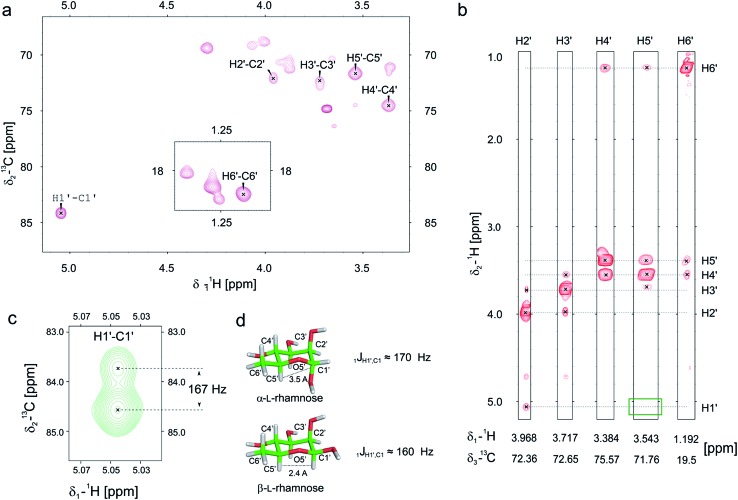
Determination of the EarP rhamnosylation mechanism *via* NMR. (a) Zoomed in view of the sugar resonance region of the ^13^C-HSQC of rhamnosylated EF-P. The assignment is based on a ^13^C-edited NOESY-HSQC (exemplary strips are shown in panel b). Unassigned peaks at around 70 ppm and 18 ppm are the resonances of EF-P's threonine H_β_/C_β_ and methyl groups, respectively. (b) Strips of the ^13^C-edited NOESY-HSQC to illustrate the lack of an observable NOE between H1′ and H5′ (green rectangle), which confirms that the rhamnose adopts an α-configuration, when bound to EF-P. (c) H1′–C1′ resonance of EF-P rhamnose from an undecoupled ^13^C-HSQC to derive the ^1^ *J*_CH_ coupling. The resulting coupling of 167 Hz indicates an α-configuration of the sugar.^21,22^ (d) Stick representations of α-l-, and β-l-rhamnose.

Previously we and others demonstrated that mono-rhamnosylated EF-P-Arg32 in the EarP-arginine phylogenetic subfamily is essential to efficiently alleviate ribosome stalling at polyproline stretches.^14–16^ However, nothing is known about the anomeric configuration of the attached sugar. Knowledge about steric configuration is important to understand how the modification contributes to the stabilization of the CCA-end of the P-site prolyl-tRNA and to classify EarP either as a retaining or inverting glycosyltransferase.^19,20^ Notably, the activated sugar substrate is dTDP-β-l-rhamnose. In order to determine the configuration after glycosylation, we employed ^13^C-edited NOESY-HSQC to assign the sugar resonances (Fig. 2a and b). ^1^ *J*_CH_ couplings can inform about the configuration of the anomeric carbon. An equatorial position of H1′ (α-anomer) would result in a coupling of around 170 Hz, whereas ≤160 Hz would indicate an axial position (β-anomer).^21,22^ An undecoupled ^13^C-HSQC gave rise to a coupling of 167 Hz, clearly indicating an α-configuration on the protein acceptor EF-P (Fig. 2c and d). This was confirmed by the absence of an observable NOE between H1′ and H5′ (Fig. 2b). If H1′ was in the axial position a strong NOE should be visible. The change of configuration at the anomeric center from dTDP-β-l-rhamnose to Arg-α-l-rhamnose during the glycosylation reaction identifies EarP to be an inverting glycosyltransferase.

### Synthesis of the α-rhamnosylated arginine containing hapten

**Fig. 3:**
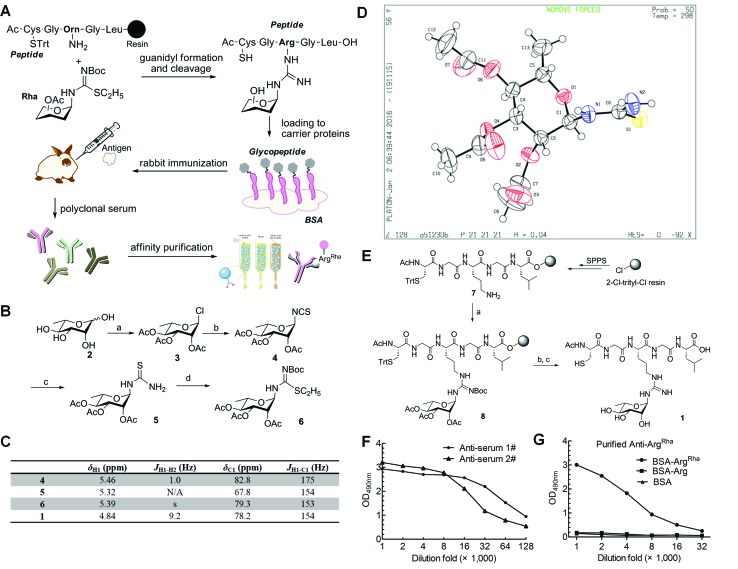
Synthesis of mono-Arg^Rha^ peptide and antibody generation. (A) Work-flow of antibody generation: in the first step an Arg^Rha^ containing glycopeptide was synthesized *via* guanidyl formation, cleavage and subsequent coupling to bovine serum albumin (BSA). The resulting glycoconjugate was used to immunize rabbits and accordingly to collect crude sera containing polyclonal antibodies against Arg^Rha^. Using a two-step affinity chromatography technique we finally purified a highly sensitive and specific polyclonal *anti*-Arg^Rha^ antibody. Trt = trityl; Boc = *tert*-butoxycarbonyl. (B) Synthesis of building block **6**. Reagents and conditions: (a) acetyl chloride, r.t., 2 days, 85%; (b) KSCN, TBAI, and CH_3_CN, reflux, 3 h, 70%; (c) NH_3_, and THF, 1 h, 99%; (d) EtI, and MeOH, reflux, 3 h; then Boc_2_O, Et_3_N, and CH_2_Cl_2_, 75%. (C) NMR spectroscopic characterization of compounds **4**, **5**, **6** and **1**. (D) Single crystal structure of compound **5**. (E) Solid-phase synthesis of mono-Arg^Rha^ peptide **1**. Reagents and conditions: (a) TEA, DMF, AgNO_3_, and **6** (3 eq.), r.t.; (b) 5% NH_2_NH_2_ in DMF; (c) 5% TIPS in TFA. (F) ELISA analysis of two batches of crude *anti*-sera. The crude *anti*-sera immunized by the BSA-glycoconjugate can recognize Arg^Rha^ with high affinity. *anti*-Serum 1# and *anti*-serum 2# were successively diluted up to 128 000 fold and subjected to indirect ELISA experiments against the BSA-glycoconjugate. (G) ELISA analysis of purified *anti*-Arg^Rha^. Purified *anti*-Arg^Rha^ can recognize Arg^Rha^ with high specificity. The purified antibody was successively diluted up to 32 000 fold and subjected to indirect ELISA experiments against the BSA-glycoconjugate (BSA-Arg^Rha^) and BSA carrying the non-glycosylated peptide (BSA-Arg).

Having solved the configuration of the rhamnose moiety attached to EF-P-Arg32 we were encouraged to raise specific antibodies against the modification employing an α-rhamnosylated arginine containing peptide, for immunization (Fig. 3a). Such a modification-specific antibody would be a useful tool to investigate EarP mediated rhamnosylation *in vivo* and *in vitro* but might also help in the identification of further arginine rhamnosylated proteins from diverse organisms.

Based on previous work,^23,24^ we chose a strategy for glycopeptide synthesis that involves direct silver-promoted glycosylation between an *S*-alkyl-isothiourea and the amine of the amino acid side chain on the solid phase. First, we synthesized the key building block, *N*-glycosyl-*S*-alkyl-isothiourea **6**, starting from l-rhamnose **2** (Fig. 3b): glycosyl chloride **3** in the desired configuration was obtained using well established procedures (85% yield).^25,26^ Subsequently, **3** was treated with potassium thiocyanate (KSCN) and tetrabutylammonium hydrogen iodide (TBAI) in anhydrous acetonitrile to get glycosyl isothiocyanate **4** (70% yield).^27^ Next, glycosyl thiourea **5** was prepared *via* ammoniation of **4** in tetrahydrofuran (99% yield).^28^ Finally, a two-step, one-pot procedure converted **5** into **6** in the presence of ethyl iodide and *tert*-butoxycarbonyl anhydride (75% yield).^29^ Taken together from **2** to **6** we ended up with an efficiency of about 44%. The configuration of the attached rhamnose in the hapten depends on the stereochemistry of the key intermediate compounds **5** or **6**. NMR-HSQC showed that the ^1^ *J*_CH_ coupling underwent a change from 174 Hz to 154 Hz (Fig. 3c) when compound **4** was converted into **5**. Thus we determined the anomeric carbon configuration of compound **5**. A single crystal was obtained *via* slow evaporation of a dichloromethane/*n*-hexane solution at room temperature (Fig. 3d). With this (*N*-(2,3,4-tri-*O*-acetyl-6-deoxy-α-l-manno-pyranos-1-yl)thiourea) in hand, we could show unambiguously that the rhamnose moiety is attached in an α configuration, being consistent with rhamnosylated EF-P. All of the intermediates were fully characterized using ^1^H-NMR, ^13^C-NMR, and HR-Q-TOF-MS (Fig. 3c and ESI†).

To synthesize the hapten glycopeptide **1** with the rhamnose moiety in the α-configuration from building block **6**, we chose an on-resin glycosylation strategy (Fig. 3e): to obtain the linear peptide we used 9-fluorenylmethyloxycarbonyl (Fmoc) SPPS standard procedures with Fmoc-Orn(Alloc)-OH as the precursor for the Arg^Rha^ residue. A 2-chlorotrityl resin acted as the solid support. Subsequent to the peptide assembly, the Alloc group was removed in the presence of tetrakis(triphenylphosphine)palladium (**0**) to get compound **7** on-resin.^30–34^ Then the on-resin glycopeptide **8** was synthesized with a silver-promoted solid-phase glycosylation between the free amino group of **7** and the key building block **6**. Next, the rhamnose moiety was deacetylated with 5% NH_2_NH_2_ in dimethylformamide (DMF).^35^ Subsequently the resin was treated with 5% triisopropylsilane (TIPS) in trifluoroacetic acid (TFA) to release the glycopeptide **1** which was further purified *via* preparative reverse-phase HPLC. We calculated from resin loading that the total yield of isolated **1** was 28%, manifesting a good efficiency for the on-resin glycosylation process.^36–38^ All of the key intermediates were monitored using analytical HPLC and characterized using HR-Q-TOF-MS (Fig. S1†). The final peptide – Cys–Gly–Arg(Rha)–Gly–Leu – was characterized using 1D-NMR, 2D-NMR, and HR-Q-TOF-MS.

### Generation and purification of a rhamnosyl arginine specific primary antibody

To raise the high affinity Arg^Rha^ specific antibody (*anti*-Arg^Rha^), the hapten was conjugated to BSA as carrier protein *via* the free N-terminal sulfhydryl group distal from the arginine rhamnosyl side chain (Fig. 3a). The resulting BSA-glycoconjugate was injected into rabbits to raise polyclonal antibodies targeting the Arg^Rha^ moiety.^39,40^ After the third immunization, the crude *anti*-sera were collected and their specificity was monitored by employing an enzyme-linked immunosorbent assay (ELISA) analysis with horseradish peroxidase linked *anti*-rabbit IgG. The antibodies from two batches of crude *anti*-sera from two immunized rabbits were found to bind robustly and specifically to the BSA-glycoconjugate with high titers, showing strong immune reactivity even after 128 000 fold dilution (Fig. 3f and S3†).

To purify *anti*-Arg^Rha^ from the crude rabbit *anti* sera, in a first step we used a Protein A Sepharose 4 column (Amersham Biosciences). In a second purification step two agarose columns coupled with BSA or BSA carrying the non-glycosylated “naked” pentapeptide (H-CGRGL-OH) were used to exclude cross-reactivity. Taken together, these two steps resulted in a 95% pure *anti*-Arg^Rha^ antibody (Fig. S4 and S5†) showing a significantly improved specificity against the glycoconjugated BSA compared to the crude *anti*-sera (Fig. 3g and S6†).

### *anti*-Arg^Rha^ allows sensitive and specific detection of endogenous EF-P^Rha^

**Fig. 4:**
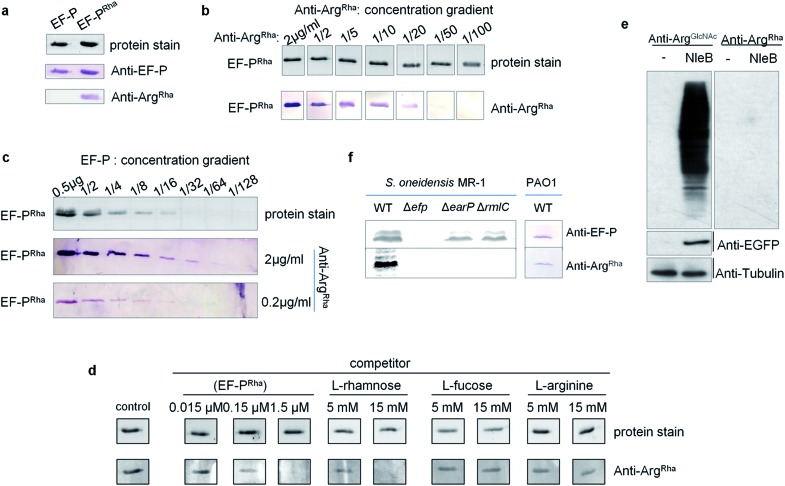
Sensitivity and specificity analysis of *anti*-Arg^Rha^ against EF-P^Rha^. (a) The *anti*-Arg^Rha^ antibody specifically recognizes EF-P^Rha^. Immunodetection of purified EF-P both unmodified (EF-P) and rhamnosylated (EF-P^Rha^) using *anti*-EF-P and *anti*-Arg^Rha^. 0.5 μg of purified EF-P was subjected to SDS-PAGE and subsequent Western Blot analysis with 0.2 μg ml^–1^ *anti*-EF-P or *anti*-Arg^Rha^ respectively. (b) Immunodetection of EF-P^Rha^ when *anti*-Arg^Rha^ was successively diluted. (c) Immunodetection of EF-P^Rha^ when EF-P^Rha^ was successively diluted and *anti*-Arg^Rha^ was used in concentrations of 2 μg ml^–1^ or 0.2 μg ml^–1^. (d) Cross-reactivity analysis of *anti*-Arg^Rha^ against l-rhamnose, l-fucose and l-arginine. 0.5 μg of purified EF-P^Rha^ were subjected to SDS-PAGE and subsequent Western Blot analysis using 0.2 μg ml^–1^ *anti*-Arg^Rha^. *anti*-Arg^Rha^ was preincubated with varying concentrations of EF-P^Rha^, l-rhamnose, l-fucose and l-arginine. Buffer only served as a control. (e) *anti*-Arg^Rha^ cannot detect Arg^GlcNAc^. 293T cells were transfected with mock vector or pCS2–EGFP–NleB plasmids. Western Blot analysis of total cell lysates using either *anti*-Arg^GlcNAc^ or *anti*-Arg^Rha^. *anti*-EGFP and *anti*-tubulin served as a control. (f) Detection of EF-P^Rha^ from *S. oneidensis* MR-1 lysates of wildtype (WT) and different mutant strains lacking *efp* (Δ*efp*) the glycosyltransferase EarP (Δ*earP*) or interfering with dTDP-β-l-rhamnose biosynthesis (Δ*rmlC*). *P. aeruginosa* PAO1 WT crude lysates served as an additional *in vivo* control. Approximately, 10^8^ cells were used per lane.

Having the *anti*-Arg^Rha^ at hand, we tested whether we can detect the EF-P rhamnose modification. Therefore we used 0.5 μg of purified EF-P which was modified *in vivo* (EF-P^Rha^) employing the enzymatic activity of EarP. Unmodified EF-P served as a negative control. As expected, an EF-P specific antibody (*anti*-EF-P) detected both protein variants with no difference in signal intensity. By contrast *anti*-Arg^Rha^ specifically targeted only the EF-P Arg^Rha^ modification and no signal occurred in the lane with unmodified EF-P (Fig. 4a). The amino acid context of the arginine rhamnosylation site in EF-P is Ser_30_–Gly_31_–Arg_32_(Rha)–Asn_33_–Ala_34_ and thus significantly differs from the peptide Cys–Gly–Arg(Rha)–Gly–Leu, previously used to raise *anti*-Arg^Rha^. Thus, we conclude that our antibody recognizes Arg^Rha^ irrespective of the adjacent amino acid residues.

Next we assessed the detection limits of *anti*-Arg^Rha^ by using varying concentrations of either EF-P^Rha^ or *anti*-Arg^Rha^ (Fig. 4b and c). When keeping the EF-P^Rha^ concentration constant at 25 μg ml^–1^ (1.25 μM), the signal intensity progressively decreased starting from 2 μg ml^–1^ *anti*-Arg^Rha^ until no further detection was possible at an antibody concentration of 0.04 μg ml^–1^. When keeping the *anti*-Arg^Rha^ concentration constant at 2 μg ml^–1^, 15 ng of EF-P^Rha^ were efficiently detected.

To further prove the specificity against Arg^Rha^ we performed a Western Blot based competition assay in which our antibody was preincubated with various concentrations of l-rhamnose, l-fucose, or l-arginine. Pre-added EF-P^Rha^ served as specific competitor and completely prevented detection of EF-P already at concentrations of 1.5 μM (Fig. 4d). On the contrary even 15 mM of l-arginine or l-fucose could not decrease the signal intensity. At this concentration only l-rhamnose abolished the EF-P^Rha^ signal and therefore constitutes a competitor that is around 10 000 times less effective than EF-P^Rha^ (Fig. 4d and S7†). To examine whether the *anti*-Arg^Rha^ antibody shows cross reactivity towards other types of arginine *N*-glycosylation, we prepared lysates from 293T cells ectopically expressing NleB. As expected arginine GlcNAcylation could be detected using *anti*-Arg^GlcNAc^ ^9,24^ but no signal occurred when using *anti*-Arg^Rha^ (Fig. 4e). Taken together, our antibody can be regarded as highly sensitive and specific against arginine rhamnosylation.

We ultimately asked whether we can visualize endogenous arginine rhamnosylated proteins from crude cell lysates. From our sensitivity analysis we calculated that it was possible to detect single Arg^Rha^ with about 100 copies per cell when subjecting 10^8^ cells and 2 μg ml^–1^ *anti*-Arg^Rha^ to Western Blot analysis. Rich media exponentially growing *E. coli* EF-P carry about 10 000 copies of EF-P per cell^41^ and therefore it should be possible to detect the modified protein. As *Enterobacteriales* modify EF-P with (*R*)-β-lysine^42–44^ we used *S. oneidensis* which naturally employs EarP mediated rhamnosylation. Whereas we could readily identify EF-P in wildtype cells, mutants lacking either *efp* or *earP* gave no signal (Fig. 4f). Similarly, we could not detect EF-P rhamnosylation in a strain Δ*rmlC* that cannot produce the EarP substrate for glycosylation – dTDP-β-l-rhamnose. We used *P. aeruginosa* PAO1 crude cell lysates to test the activity of the *anti*-Arg^Rha^ antibody in another species and detected a single band (Fig. 4f). The band was verified to be EF-P in a parallel Western Blot, yielding a signal at the same height, by use of a *S. oneidensis anti*-EF-P antibody. Thus our *anti*-Arg^Rha^ represents a potent tool to detect EF-P rhamnosylation in diverse species.

## Conclusion

We recently demonstrated the use of a high affinity *anti-N*-acetyl glucosaminyl arginine antibody (*anti*-Arg^GlcNAc^) to monitor the glycosylation of human death receptor domains mediated by NleB during EPEC infection.^9,24^ Similarly, *anti*-Arg^Rha^ represents a novel tool to diagnose infections caused by pathogens such as *P. aeruginosa* ^14,15^ or *N. meningitidis*.^16^ Ultimately, our *anti*-Arg^Rha^ might allow us to identify further arginine-rhamnosylated proteins from diverse species. This in turn might help to unveil novel antimicrobial targets and contribute to the task of overcoming the increasing problem of multi resistance. In this regard, it is indispensable to understand the mode of action of arginine dependent glycosyltransferases as they appear to be involved in pathogenicity development. However, our knowledge of *N*-linked glycosylation is so far mainly restricted to asparagine. The stereospecific outcome of the glycosylation reaction is a major characteristic of its molecular mechanism. By determining the α-anomeric nature of the rhamnosyl moiety on EF-P and with this the inverting mode of glycosyl transfer mediated by EarP, we made the first step to elucidating the catalysis of this novel type of glycosyltransferase. Our finding might also help to further understand how the sugar participates in stabilizing the CCA-end of the P-site prolyl-tRNA and thus contributes to the rescue of polyproline dependent ribosome arrest situations.

## Author contributions

X. L. performed the organic synthesis, analysis of NMR and single crystal structures of small molecules, generated the *anti*-Arg^Rha^ antibody and wrote the manuscript. R. K. performed the confirmation of antibody specificity, produced proteins for the NMR-analysis of rhamnosylated EF-P and wrote parts of the manuscript. J. M., B. S. and J. H. performed the NMR determination of the anomeric carbon configuration of attached rhamnose and wrote parts of the manuscript. Y.-L. L. assisted in the organic synthesis. Y. Z., Q.-Y. W. and F. Y. assisted in the organic synthesis and antibody generation. X. P. and S. L. assisted in the confirmation of antibody specificity. J. L. performed the confirmation of antibody specificity, contributed to study design and wrote the manuscript. K. J. and H.-G. H. contributed to study design and assisted in modification of the manuscript.
