# Supplementary material for: Resolving the α-glycosidic linkage of arginine-rhamnosylated translation elongation factor P triggers generation of the first ArgRha specific antibody
Source: Chem Sci. 2016 Jul 21;7(12):6995–7001. doi: 10.1039/c6sc02889f (PMC5363779; doi:10.1039/c6sc02889f)
Supplement: Supplementary file 1 [file SC-007-C6SC02889F-s001.pdf]

Electronic Supplementary Material (ESI) for Chemical Science.
This journal is © The Royal Society of Chemistry 2016

Supporting information

Resolving the α-Glycosidic Linkage of Arginine-Rhamnosylated Translation Elongation Factor P (EF-P) Triggers Generation of the First Arg^{Rha} Specific Antibody **

Xiang Li,[†a] Ralph Krafczyk,[†b] Jakub Macošek,[c] Yu-Lei Li,[ad] Yan Zou,[a] Bernd Simon,[c] Xing Pan,[e] Qiu-Ye Wu,[a] Fang Yan,[d] Shan Li,[e] Janosch Hennig,[c] Kirsten Jung,[b] Jürgen Lassak*[b] and Hong-Gang Hu*[a]

a Department of Organic Chemistry, School of Pharmacy, Second Military Medical University, Shanghai 200433, China

b Department of Biology I, Microbiology, Ludwig Maximilians-Universität München, Munich, Germany

Center for Integrated Protein Science Munich, Ludwig-Maximilians-Universität München, Munich, Germany

c Structural and Computational Biology Unit, EMBL Heidelberg, Heidelberg 69117, Germany

d School of Pharmacy, Wei Fang Medical University, Shandong 261053, China

e Institute of infection and immunity, Taihe hospital, Hubei university of medicine, Shiyan, Hubei 442000

(China)

[†]: These authors contributed equally to this work.

*Email: juergen.lassak@lmu.de; huhonggang_fox@msn.com

Table of Contents

1. General Information

1.1 Materials

1.2 HPLC

1.3 Mass spectrometry and NMR of small moleculars

2. Nuclear magnetic resonance spectroscopy

3. Chemical Synthesis and X-ray crystallography

3.1 2,3,4-Tri-*O*-acetyl-6-deoxy-*α*-L-mannopyranosyl chloride (**3**)

3.2 2,3,4-Tri-*O*-acetyl-6-deoxy-*α*-L-mannopyranosyl isothiocyanate (**4**)

3.3 *N*-(2,3,4-Tri-*O*-acetyl-6-deoxy-*α*-L-mannopyranos -1-yl) thiourea (**5**)

3.4 1-(tert-Butoxycarbonyl)-3-(2,3,4-Tri-*O*-acetyl-6-deoxy-*α*-L-mannopyranos-1-yl)-2-ethyl-isothiourea (**6**)

3.5 Synthesis of the title hapten glycopeptide **1**

3.6 X-ray crystallography of compound **5**

4. Methods for antibody generation and purification

4.1 Conjugation of glycopeptides to BSA

4.2 Rabbit immunization

4.3 Affinity purification of antibodies

4.4 ELISA of crude anti-*Arg*-*Rha* antiserum

5. Specificity of the anti-$\mathit{Arg}^{\mathit{Rha}}$ for EF-P^{Rha}

5. 1 Bacterial strains and growth conditions

5.2 Construction of plasmids for protein overproduction.

5.3 Production and purification of unmodified and rhamnosylated EF-P.

5.4 Immunodetection analysis of *S. oneidensis* EF-P

6. NMR spectrum

7. References

1. General Information

1.1 Materials

All reagents and solvents were purchased from Acros Organics, Alfa Aesar or Sinopharm Chemical Reagent Co. Ltd and were purified when necessary. THF was distilled from sodium/benzophenone ketyl before use. DMF was distilled under reduced pressure from sodium sulfate and stored in flask containing 4 Å molecular sieves. Et_3N and CH_2Cl_2 were distilled from calcium hydride immediately prior to use. All organic extracts were dried over sodium sulfate and concentrated under rotary evaporator. All other commercially obtained reagents and solvents were used directly without further purification. TLC was performed on plates pre-coated with silica gel 60 F_{254} (250 layer thickness). Flash column chromatographic purification of products was finished using forced-flow chromatography on Silica Gel (300-400 mesh). Visualization was accomplished with 5% (v/v) H_2SO_4 in EtOH, UV light, and/or phosphomolybdic acid (PMA) solution.

1.2 HPLC

Protein A chromatography was carried out on an ÄKTA Explorer chromatographic system from Amersham Biosciences with built-in UV, pH and conductivity detectors to monitor column effluent.

Analytical HPLC was run on a SHIMADZU (Prominence LC-20AD) instrument using an analytical column (Grace Vydac "Protein & Peptide C18", 250 X 4.6 mM, 5 μm particle size,

flow rate 1.0 mL/min, rt). Analytical injections were monitored at 214 nm, 254 nm. Semi preparative HPLC was run on a SHIMADZU (LC-6A) instrument using a semi preparative column (Grace Vydac "Peptide C18", 250 X 10 mM, 10 μm particle size, flow rate 4 mL/min). Solution A was 0.1% TFA in water, and solution B was 0.1% TFA in MeCN. Gradient A: A linear gradient of 10% to 10% B over 2 min, then a linear gradient of 10% to 90% B over 25 min. Gradient B: A linear gradient of 1% to 1% B over 3 min, then a linear gradient of 1% to 35% B over 25 min.

1.3 Mass spectrometry and NMR of small moleculars

^{1}H-NMR and ^{13}C-NMR spectra were obtained on a Bruker Avance 300 MHz or 600 MHz NMR Spectrometer. The chemical shifts of protons are given on the *δ* scale, ppm, with tetramethylsilane (TMS) as the internal standard. HR-Q-TOF-MS was measured on an Agilent 6538 UHD Accurate Mass Q-TOF LC/MS mass spectrometer.

2. Nuclear magnetic resonance spectroscopy

In order to determine the configuration of rhamnose when bound to EF-P, the rhamnosylated protein was expressed in M9 minimal medium with ^{13}C-Glucose and ^{15}N-ammonium chloride as sole carbon and nitrogen source, respectively. Triple resonance experiments[1] were used to assign backbone chemical shifts of the protein. The rhamnose moiety was assigned, using a sensitivity-enhanced ^{13}C-edited NOESY-HSQC with simultaneous evolution of ^{13}C and ^{15}N during t_2[2]. Three undecoupled, sensitivity-enhanced ^{13}C-HSQC[3] were acquired to obtain the $^1J_{CH}$ coupling of the anomeric carbon to its proton (H1'-C1'). One without ^{13}C decoupling in the ^{1}H direct dimension during acquisition to obtain the $^1J_{CH}$ coupling constant resolved in the ^{1}H dimension with 4096 points (160 ms acquisition time) for sufficient resolution to allow reliable fitting of the coupling constant. The second and third without ^{1}H decoupling during ^{13}C chemical shift evolution to resolve the $^1J_{CH}$ coupling in the ^{13}C indirect dimension was acquired with 3584 points (63.6 ms) to obtain sufficient resolution and with 600 points (10.7 ms) to not resolve ^{13}C-^{13}C coupling (see figure 2c). In order to acquire the ^{13}C dimension with large spectral width to include the methyl, aliphatic, sugar, and aromatic regions with sufficient carbon decoupling, but without sideband artifacts we employed low-power broadband heteronuclear decoupling based on optimal control theory[4]. All spectra were processed using NMRPipe[5] with a gaussian window function and zero filling. Peak detection and fitting of the coupling constant has been used with in-built functions in NMRPipe

(nlinLS) using gaussian fitting. The coupling constants derived from fitting the three spectra are consistent.

3. Chemical synthesis

3.1 2,3,4-Tri-O-acetyl-6-deoxy-α-L-mannopyranosyl chloride (3)

Acetyl chloride (60.0 mL, 843.6 mmol) was added dropwise into a round flask containing L-Rhamnose (**2**, 25.0 g, 152.4 mmol). The mixture was stirred for 48 h under Ar. Dichloromethane (300 mL) was added and the organic layer was washed with water (3*100 mL) and then saturated sodium bicarbonate (3*100 mL). The organic layer was dried over sodium sulfate and concentrated. The residue was purified by column chromatography eluting with 4/1 petroleum ether/ethyl acetate to give **3** as a white foam (39.9 g, 85 %). ^{1}H NMR (300 MHz, $CDCl_3$): δ 5.91 (d, J = 0.8 Hz, 1 H), 5.56 (dd, J = 10.2 Hz, 4.2 Hz, 1 H), 5.37 (q, J = 1.7 Hz, 1 H), 5.15 (t, J = 10.1 Hz, 1 H), 4.17- 4.08 (m, 1 H), 2.15 (s, 3 H), 2.06 (s, 3 H), 1.98 (s, 3 H), 1.26 (d, J = 6.4 Hz, 3H). ^{13}C NMR (75 MHz, $CDCl_3$): δ 169.9, 169.8, 169.7, 89.0, 71.9, 70.3, 69.4, 67.7, 20.8, 20.8, 20.6, 17.1.

3.2 2,3,4-Tri-O-acetyl-6-deoxy-α-L-mannopyranosyl isothiocyanate (4)

A mixture of tetrabutylammonium iodide (9.18 g, 25.0 mmol), KSCN (4.85 g, 50.0 mmol), and molecular sieve (4 Å, 20 g) in dry acetonitrile (200 mL) was stirred at room temperature under argon for 2 h. Then compound **3** (7.70 g, 25.0 mmol) was added to the solution and the mixture was refluxed for another 3 h. Then the mixture was filtered, and the filtrate was concentrated *under vacuum*. The residue was purified by column chromatography with 4/1 petroleum ether/ethyl acetate to yield compound **4** (5.80 g, 70 %) as a white solid. ^{1}H NMR

(300 MHz, $CDCl_3$): δ 5.46 (d, J = 1.0 Hz, 1 H), 5.29- 5.24 (m, 2 H), 5.10- 5.03 (m, 1 H), 4.01- 3.96 (m, 1 H), 2.15 (s, 3 H), 2.06 (s, 3 H), 1.99 (s, 3 H), 1.26 (d, J = 6.2 Hz, 3 H). ^{13}C NMR (75 MHz, $CDCl_3$): δ 170.8, 170.6, 170.6, 169.2, 143.3, 82.8, 77.2, 74.0, 71.7, 67.9, 61.7, 56.1, 23.3, 20.7, 20.6, 20.6. HR-Q-TOF-MS: calcd. for $C_{13}H_{17}NNaO_7S^+$ $[M+Na]^+$ m/z, 354.0618; found, 354.0620.

3.3 N-(2,3,4-Tri-O-acetyl-6-deoxy-α-L-mannopyranos -1-yl) thiourea (5)

Gaseous ammonia was passed through a solution of **4** (3.31 g, 10 mmol) in anhydrous THF (50 mL) for 1 h. Compound **5** was obtained as a white solid after removal of solvent *in vacuum* (3.3 g, 99 %) and used in the next step without further purification. ^1H NMR (300 MHz, $CDCl_3$): δ 7.72 (s, 1 H), 6.86 (s, 2 H), 5.45-5.29 (m, 3 H), 5.08 (t, J = 9.5 Hz, 1 H), 3.91-3.86 (m, 1 H), 2.14 (s, 3 H), 2.03 (s, 3 H), 1.99 (s, 3 H), 1.22 (d, J = 6.2 Hz, 3 H). ^{13}C NMR (75 MHz, $CDCl_3$): δ 184.4, 170.1 (2 C), 167.0, 80.9, 70.5, 68.7, 67.8, 65.9, 20.9, 20.8, 20.8, 17.4. HR-Q-TOF-MS: calcd. for $C_{13}H_{20}N_2NaO_7S^+$ $[M+Na]^+$ m/z, 371.0883; found, 371.0887.

3.4 1-(tert-Butoxycarbonyl)-3-(2,3,4-Tri-O-acetyl-6-deoxy-α-L-mannopyranos -1-yl) -2-ethylisothiourea (6)

A solution of **5** (2.8 g, 8.0 mmol) and EtI (0.76 mL, 9.6 mmol) in 40 mL of anhydrous CH_3OH was stirred under reflux for 4 h. The solvent was removed *in vacuo* and the residue was desolved in dry DCM (50 mL) by adding Et_3N (2.24 mL, 16.0 mmol), $(Boc)_2O$ (1.96 g,

9.0 mmol) and catalytic amount of DMAP. After the mixture was stirred at room temperature overnight, it was washed with water and brine. The organic layer was dried by Na_2SO_4 and concentrated under vacuum. The residue was purified by flash chromatography eluting with 5:1 petroleum ether/acetone and further crystallized from ether to yield compound **6** (2.86 g, 75 %) as a white solid. ^{1}H NMR (600 MHz, $CDCl_3$): δ 5.39 (s, 1 H), 5.17 (s, 1 H), 5.04- 5.01 (m, 2 H), 3.62- 3.57 (m, 1 H), 3.13- 3.02 (m, 2 H), 2.24 (s, 3 H), 2.05 (s, 3 H), 1.98(s, 3 H), 1.45 (s, 9 H), 1.30- 1.25 (m, 6H). ^{13}C NMR (150 MHz, $CDCl_3$): δ 170.3, 170.2, 169.9, 80.1, 79.3, 72.1, 71.1, 70.0, 69.4, 28.1 (3 C), 25.5, 20.8 (2 C), 20.6, 17.5, 13.9. ESI MS: calcd. for $C_{20}H_{33}N_2O_9S^+$ $[M+H]^+$ m/z, 477.1901; found, 477.1909.

3.5 Synthesis of the title hapten glycopeptide 1

After a mixture of 2-Cl-trityl-Cl resin (400.0 mg, active Cl, 0.20 mmol), Fmoc-Leu-OH (354.0 mg, 1.0 mmol) and DIEA (365.0 μL) in DMF (5 mL) was shaken on a vortex mixer at rt overnight, the resin was filtered off and washed several times with MeOH, DCM and DMF. The Leu-linked resin was then used for the construction of full length peptide/glycopeptides. The protocols employed were: deprotection of Fmoc with 20% piperidine in DMF (15 min) and peptide coupling using 5 eq. of amino acid, 4.5 eq. of HCTU and 10 eq. of DIEA. All coupling reactions were set to perform 1 h at rt. Amino acids Fmoc-Gly-OH, Fmoc-Orn(Aloc)-OH, Fmoc-Gly-OH, Fmoc-Cys-OH, and AcOH were sequentially installed to construct peptide after removal of allyloxycarbonyl group on the resin. Then, a solution of compound **6**

(3 eq.), Et_3N (10 eq.) and $AgNO_3$ (3 eq.) in 5 mL of anhydrous DMF was added into the resin. After the mixture was shaken for 8 h in the dark, the resultant mixture was filtered and the resin was washed thoroughly with DMF, MeOH, and DCM to compound **8**. To remove the acetyl groups from the glycopeptide on the resin, the peptide-loaded resin was treated with a mixture of NH_2-NH_2 in DMF (5 %, 10 mL) for 10 h at rt. The resin was filtered off and washed thoroughly with DMF, H_2O, MeOH, and DCM. Then, the peptide-loaded resin was treated with a mixture of TIPS/TFA (5:95, 10 mL) for 2 h at rt. The resin was filtered off and washed with TFA. The washings were combined and condensed in vacuum to give crude **1**. The crude product was dissolved in water and purified by HPLC (conditions: Grace Vydac "Peptide C18", 250 X 10 mM, 10 μm particle size, suitable ratio of acetonitrile-0.1%TFA in water-0.1%TFA, 4 mL/min) to give **1** (18.0 mg, isolated yield 27 %) as a white solid. ^1H NMR (600 MHz, DMSO-*d6*) : δ 8.29-7.40 (signal of amide protons), 5.33 (s, 1 H), 4.84 (d, J = 9.2 Hz, 1H), 4.34- 4.21 (m, 3H), 3.75- 3.73 (m, 4H), 3.67 (s, 1H), 3.28- 3.25 (m, 3 H), 3.17- 3.12 (m, 3H), 2.79- 2.76 (m, 1 H), 2.71- 2.67 (m, 1 H), 2.46 (t, J = 6.4 Hz, 1H), 1.89 (s, 3 H), 1.76- 1.70 (m, 1 H), 1.64- 1.55 (m, 1 H), 1.55- 1.45 (m, 5H), 1.13 (d, J = 6.1 Hz, 3 H), 0.89 (d, J = 6.6 Hz, 3 H), 0.84 (d, J = 6.6 Hz, 3 H). ^{13}C NMR (150 MHz, DMSO-d_6): δ 173.9, 171.3, 170.4, 169.8, 168.7, 168.5, 155.4, 78.2, 73.6, 72.8, 71.2, 69.9, 55.4, 52.1, 50.2, 42.1, 41.5, 10.1, 29.0, 25.8, 24.6, 24.2, 22.8, 22.5, 21.3, 17.7. HR-Q-TOF-MS: calcd. for $C_{27}H_{49}N_8O_{11}S^+$ $[M+H]^+$ m/z, 693.3236; found 693.3246.

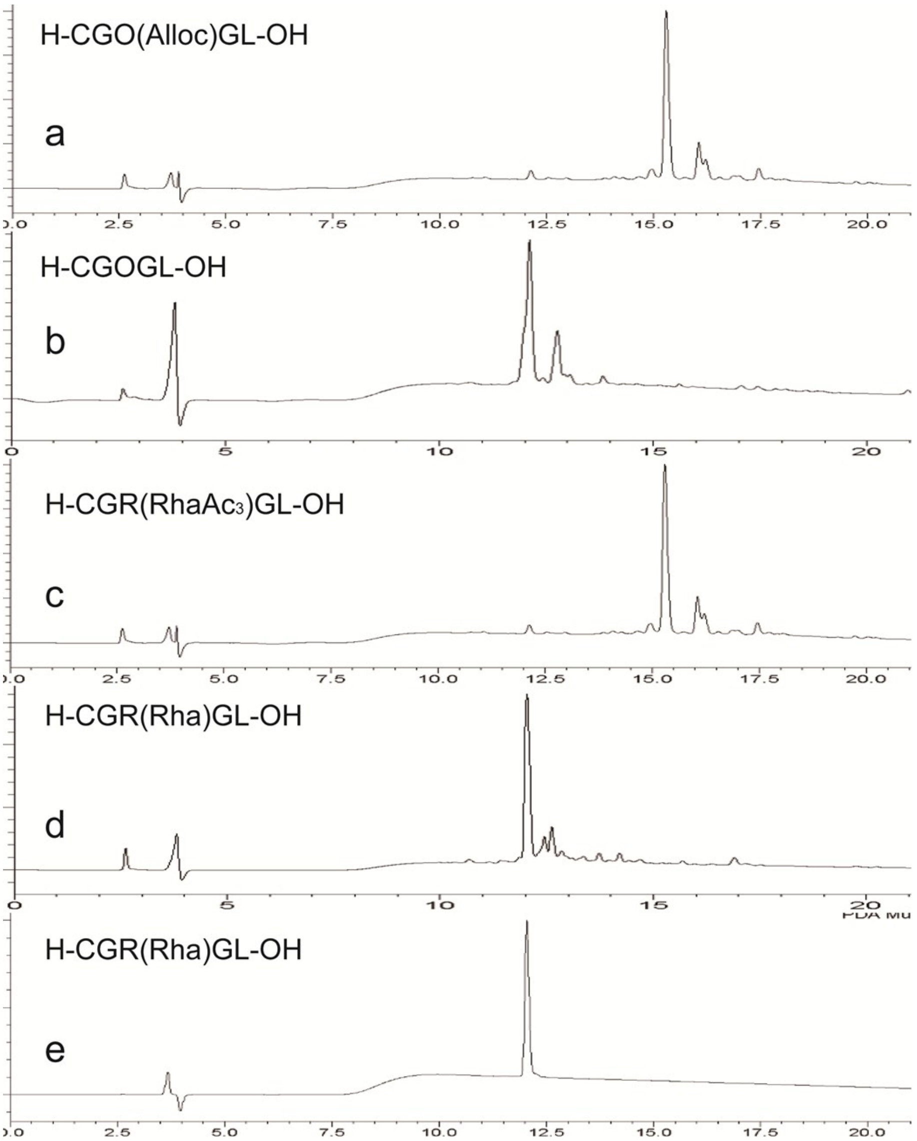

Fig S1 Analytical HPLC traces of synthetic intermediates and title compounds about gylcopeptide **1** respectively. All products were detected after TIPS/TFA cleavage of the resin. a) Full protected peptide b); Peptide **7**; c) Glycosylation of amino group of Orn with **6**/TEA/$AgNO_3$/DMF; d) NH_2NH_2 mediated removing of Ac groups, e) Analytic trace of the purified gylcopeptide **1**. Analytical HPLC was run on a SHIMADZU (Prominence LC-20AD) instrument using an analytical column (Grace Vydac "Protein & Peptide C18", 250 X 4.6 mM, 5 μm particle size, flow rate 1.0 mL/min, rt). Solution A was 0.1% TFA in water, and solution B was 0.1% TFA in MeCN. Gradient for **a-e**: A linear gradient of 10% to 10% B over 2 min, then a linear gradient of 10% to 90% B over 25 min.

3.6 X-ray crystallography of compound 5

Single crystal of compound **5** was obtained by slow evaporation of dichloromethane/n-hexane solution of **5** at room temperature. The X-ray single crystal diffraction data for **5** was collected on Bruker APEX DUO diffractometers with Mo Kα radiation (λ = 0.71073 Å) at 298 ± 2 K in the ω-2θ scanning mode. The structures were solved by direct methods using the SHELXS-97 program[6] and refined by full-matrix least-squares techniques (SHELXL-97) on F^2.

Anisotropic thermal parameters were assigned to all non-hydrogen atoms. The organic hydrogen atoms were generated geometrically. CCDC-1469830 contains the supplementary crystallographic data for this paper. These data can be obtained free of charge from the Cambridge Crystallographic Data Center via www.ccdc.cam.ac.uk/data_request/cif.

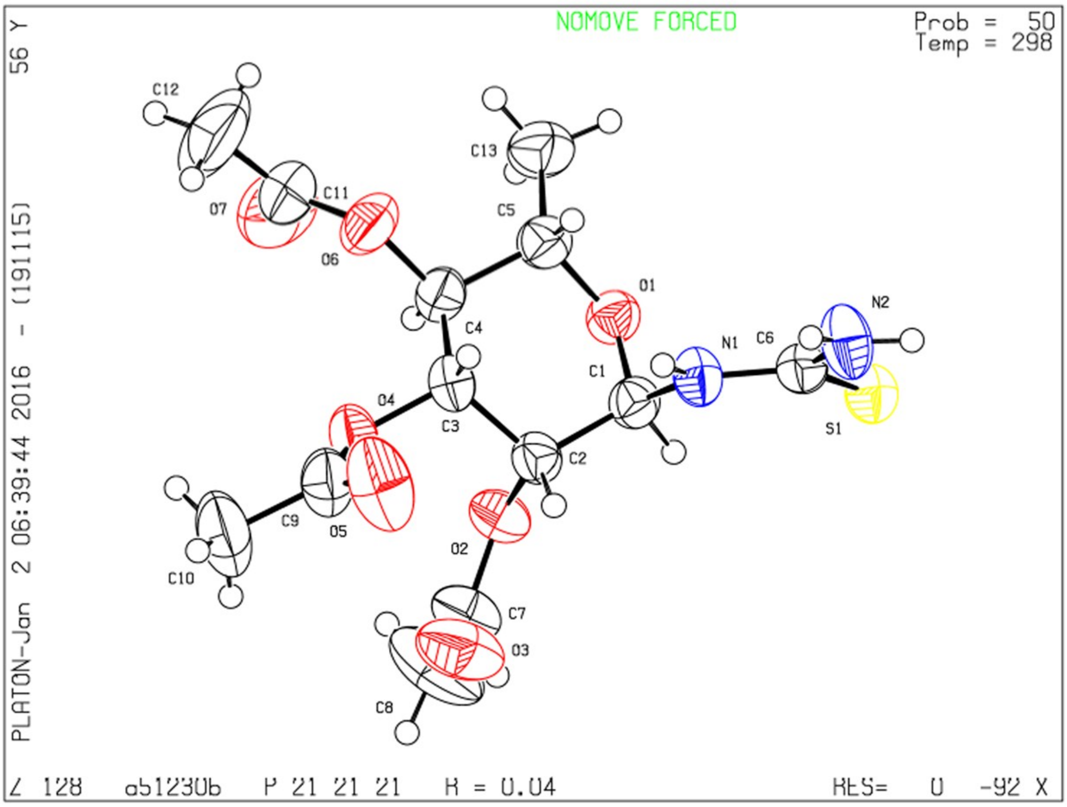

Fig S2 Single-crystal structure of Compound 5.

4. Methods for antibody generation and purification

4.1 Conjugation of glycopeptides to BSA

Glycopeptides were conjugated with Bovine serum albumin (BSA) for inoculation and ELISA analysis, respectively, using Sulfo-GMBS as the crosslinker by following the manufacturer's instruction. BSA was mixed with indicated peptides at a molecular ratio of 1:1.

4.2 Rabbit immunization

Two rabbits (male, 2- 2.5 Kg) were immunized three times at three-week intervals. P1(Rha)-BSA was injected together with the complete and incomplete form of Freund's adjuvant for the 1st and 2nd/3rd immunization, respectively. For each immunization 2 mg peptide was used. Serum samples were collected prior to immunization (pre-bleed) and 1 week after the final immunization for subsequent analyses.

4.3 Affinity purification of antibody

Firstly, the crude anti sera were purified over Protein A Sepharose 4B fast flow. Protein A chromatography was carried out on a 1.1 cm D × 10 cm H column. The column was equilibrated with 25 mM PBS, 150 mM sodium chloride, pH 7.2 buffer, washed with equilibration buffer, and eluted with 100 mM glycine-HCl, pH 2.8. The eluate was collected and neutralized with 1 M tris base. Turbidity and protein concentration were determined following neutralization of the elution pool. Next our anti sera were further purified with BSA Sepharose 4B Fast Flow & naked peptide-BSA Sepharose 4 Fast Flow. Affinity resin consisting of BSA and naked peptide-BSA immobilized onto agarose were prepared by covalently attaching BSA and naked peptide-BSA to agarose beads using CNBr-activated Sepharose 4B (GE Healthcare) according to the manufacturer’s instructions. Eluting procedure was same as that of the Protein A purification. Fractions containing rhamnosylarginine antibody were determined by SDS-PAGE and ELISA analysis.

4.4 ELISA of crude anti-Arg-Rha anti sera and purified antibody

BSA or BSA-peptides (5 μg/mL) were dissolved in coating buffer (0.032 M Na_2CO_3 and

0.068 M $NaHCO_3$, pH 9.6). 100 μL of each sample containing 0.5 μg of protein was added to a Nunc-Immuno Maxisorp 96-well plate. The plate was incubated at 4°C overnight on a nutator. The wells were then washed three times with wash buffer (phosphate buffer solution supplemented with 0.05% Tween-20) and blocked with 10% Fetal Bovine Serum (FBS) in the wash buffer (200 μL/well) for 120 min at 37°C. After removing the blocking solution, crude anti sera (including the neutralized anti sera) or purified antibody diluted in wash buffer were added to the wells (100 μL/well) and incubated for 60 min at 37°C. The neutralized crude anti sera were prior incubated with BSA for 60 min at 37°C before adding to the wells. The wells were then washed three times with the wash buffer. Goat-anti rabbit-HRP secondary antibody (1:5,000 dilution) was added to the wells (100 μL/well) and incubated for 60 min at 37°C. After extensive wash, 100 μL of fresh ELISA substrate solution (4.86 mL of 0.1 M citric acid and 5.14 mL of 0.2 M Na_2HPO_4 supplemented with 4 mg o-phenylenediamine and 15 μL of H_2O_2) was added to each well and incubated for 5-10 min at room temperature followed by addition of 50 μL of 2 M H_2SO_4 to quench the reaction. The absorbance at 490 nm was measured on a BioRad microplate reader.

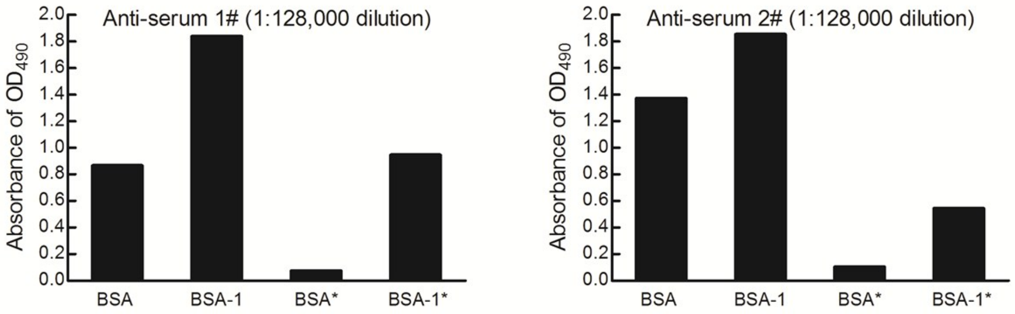

Fig S3 Anti-Arg^{Rha} crude sera immunized by BSA-glycoconjugate can recognize the arginine rhamnosylated BSA with high affnity. ELISA analysis of two batches of anti-sera immunized by glycopeptide **1**. Anti-serum 1# and anti-serum 2# were diluted 128,000 fold and subjected to indirect ELISA experiments against BSA-glycoconjugate or independent BSA. BSA-1: BSA-Arg^{Rha}, *: The anti-sera were neutralized with BSA when detecting immune reactivity. Absorbance at 490 nm is shown.

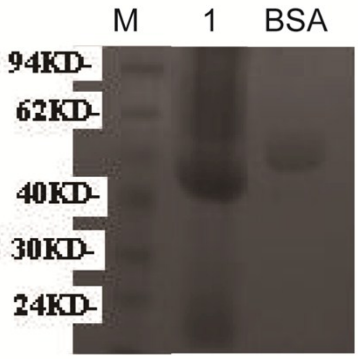

Fig S4 SDS-PAGE of the crude antibody. M: Marker, 1: Crude Anti-serum：20ul (4 mg/ml), BSA：20ug.

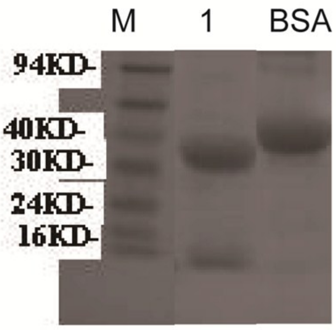

Fig S5 SDS-PAGE of the antibody after affinity purification. M: Marker, 1: Anti-Arg-Rha Rabbit IgG after specific purification：20ul (4 mg/ml), BSA：20ug.

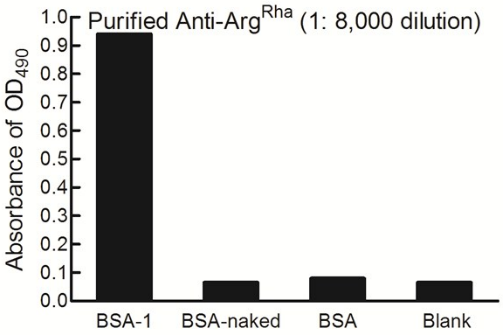

Fig S6 Purified anti-Arg^{Rha} antibody immunized by BSA-glycoconjugate can recognize the BSA-glycoconjugate with high affinity and specificity. purified anti-Arg^{Rha} antibody were diluted 8,000 fold and subjected to indirect ELISA experiments against BSA-glycoconjugate, BSA-non-glycoconjugate and independent BSA. BSA-1: BSA-Arg^{Rha} (BSA-glycoconjugate), BSA-naked: BSA-Arg (BSA-non-glycoconjugate). Absorbance at 490 nm is shown.

5. Specificity of the anti-*Arg*^{Rha} for EF-P^{Rha}

5.1 Bacterial strains and growth conditions

Strains and plasmids used in this study are listed in **Table S1**. *P. aeruginosa*, *S. oneidensis* MR-1 and *E. coli* were routinely grown in Lysogeny broth (LB) according to Miller at 30°C (for *S. oneidensis*) and 37°C (for *E. coli* and *P. aeruginosa*). When required, media were solidified by using 1.5% (w/v) agar. The optical density at 600 nm (OD600) of cultures was monitored. If necessary, media were supplemented with 30 µg/ml chloramphenicol, 50 µg/ml kanamycin sulfate, and/or 100 µg/ml ampicillin sodium salt. For promoter induction from plasmids pET SUMO, paCYC-DUET1 and derivatives 1 mM Isopropyl-*β*-D-thiogalactopyranosid (IPTG) was added to liquid medium.

5.2 Construction of plasmids for protein overproduction

Genes and gene fragments to be overexpressed were amplified from template *S. oneidensis* genomic DNA. The resulting PCR products for *efp* and *earP* were ligated into pET SUMO (Invitrogen) and pACYC-DUET1 respectively resulting in an N-terminal SUMO-tag fusion for EF-P (pET SUMO-*efp*$_{S.o.}$) and a His$_6$-tag fusion for EarP (paCYC-Duet1-e*arP*$_{S.o.}$). Routinely, chemically competent *E. coli* as strain for high-efficiency transformation[7].

5.3 Production and purification of unmodified and rhamnosylated EF-P

N-terminal His$_6$-SUMO-tagged EF-P$_{S.o.}$ was overproduced in *E. coli* BL21 (DE3) by addition of 1 mM IPTG to exponentially growing cells. After induction, cells were incubated at 16 °C

overnight. Rhamnosylated EF-$P_{S.o.}$(EF-P^{Rha}) was produced by co-overexpression of paCYC-Duet1-e*arP*$_{S.o.}$. Cells were lysed by sonication and His_6-tagged protein was purified using Ni-NTA (Qiagen) and 250 mM imidazole. The His_6-SUMO-tag was removed by incubation with 1 u/mg His_6-ulp1[8] for 16 h and pure EF-P variants were collected in the flowthrough after repeated metal chelate affinity chromatography. For use in later experiments and NMR spectroscopy, proteins were dialyzed against storage buffer. Protein concentrations were determined as described by Bradford[9].

5.4 Immunodetection analysis of S. oneidensis EF-P

Electrophoretic separation of proteins was carried out using SDS-PAGE as described by Laemmli[10]. Separated proteins were visualized in-gel using 0.5 % (v/v) 2-2-2-trichloroethanol [11] and transferred onto nitrocellulose membrane by vertical Western blotting. Antigens were detected by 0.2 µg/ml Anti-EF-P or 2 µg/ml or dilutions of Anti-Arg^{Rha} respectively. Primary antibodies (rabbit) were targeted by 0.2 µg/ml alkaline phosphatase conjugated Anti-RABBIT IgG (H&L) (GOAT) antibody (Rockland) and target proteins were visualized by addition of substrate solution (50 mM sodium carbonate buffer, pH 9.5, 0.001% (w/v) Nitro-Blue-Tetrazolium, 0,045% (w/v) 5-Bromo-4-chloro-3-indolylphosphate).

To assess the specificity of Anti-Arg^{Rha} towards rhamnosyl arginine, 0.5 µg of purified, unmodified and modified (rhamnosylated) EF-P (EF-P^{Rha}) were subjected to SDS-PAGE and Western Blot analysis as described above and targeted by 0.2 µg/ml Anti-EF-P and Anti-

Arg^{Rha} respectively.

Minimal Anti-Arg^{Rha} concentrations for detection of EF-P were determined by employing 0.5 μg of EF-P^{Rha} and decreasing amounts of Anti-Arg^{Rha} (2 μg/ml, 1 μg/ml, 0.4 μg/ml, 0.2 μg/ml, 0.1 μg/ml, 0.04 μg/ml, 0.02 μg/ml) for detection.

Antigen detection limits were determined by subjecting decreasing amounts of EF-P^{Rha} (0.5 μg, 0.25 μg, 0.125 μg, 62.5 ng, 31.25 ng, 15.6 ng, 7.8 ng, 3.9 ng) to SDS-PAGE and Western Blot analysis as described above and detection with 2 μg/ml and 0.2 μg/ml Anti-Arg^{Rha} respectively.

To investigate cross-reactivity of Anti-Arg^{Rha} against free L-arginine, L-fucose or L-rhamnose, varying concentrations of EF-P^{Rha} (0.015 μM, 0.15 μM, 1.5 μM 15 μM) and putative competitors (15 μM, 1 mM 5 mM, 15 mM) were preincubated with Anti-Arg^{Rha} prior to EF-P^{Rha} detection.

In vivo detection of EF-P and EF-P^{Rha} was carried out using total cell lysates of *Pseudomonas aeruginosa* PAO1 wildtype, *Shewanella oneidensis* MR-1 wildtype as well as *S. oneidensis* mutant strains carrying markerless inframe deletions[12] in *efp* (Δ*efp*), *earP* (Δ*earP*) and *rmlC* (Δ*rmlC*). Cells were grown to an OD600 of 1. About 15 μg total protein were subjected to SDS-PAGE and Western Blot analysis as described above. EF-P and EF-P^{Rha} were detected using 0.2 μg/ml Anti-EF-P and 2 μg/ml Anti-Arg^{Rha} respectively.

Table S1: Strains and plasmids used for EF-P detection

Strain	Genotype	Purpose	Reference
E.coli DH5αλpir	*recA*1 *gyrA* (*lacIZYA-argF*) (80d *lac* [*lacZ*] M15) *pir* RK6		[14]
E. coli BL21 (DE3)	*fhuA*2 [lon] *ompT gal* (λ DE3) [dcm] Δ*hsdS* λ DE3 = λ s*BamH*Io Δ*EcoR*I-B int::(*lacI*::P*lac*UV5::T7 gene1) i21 Δ*nin*5	Overproduction of EF-P	[15]
P. aeruginosa PAO1	*Pseudomonas aeruginosa* PAO1 wildtype		
S. oneidensis MR-1	*Shewanella oneidensis* MR-1 wildtype	*in vivo* detection of EF-P	[16]
S. oneidensis MR-1 Δ*efp*	Δ*efp* (SO_2328)	*in vivo* detection of EF-P	[13]
S. oneidensis MR-1 Δ*earP*	Δ*earP* (SO_2329)	*in vivo* detection of EF-P	[13]
S. oneidensis MR-1 Δ*rmlC*	Δ*rmlC* (SO_3160)	*in vivo* detection of EF-P	[13]
**Plasmid**	**Features**		
pET SUMO	Expression vector, Kan^r cassette, pBR322 origin, SUMO coding sequence, *lacI* coding sequence, ROP coding sequence, IPTG inducible *lac* operator		Invitrogen
pET SUMO-$efp_{S.o.}$	C-terminal His_6-Tag *efp* from *S. oneidensis* in pET SUMO		This study
paCYC-Duet1	Expression vector, Cam^r cassette, P15A replicon, *lacI* coding sequence, IPTG inducible lac operator		Novagen
paCYC-Duet1-$earP_{S.o.}$	*earP* from *S. oneidensis* in paCYC-Duet1		This study

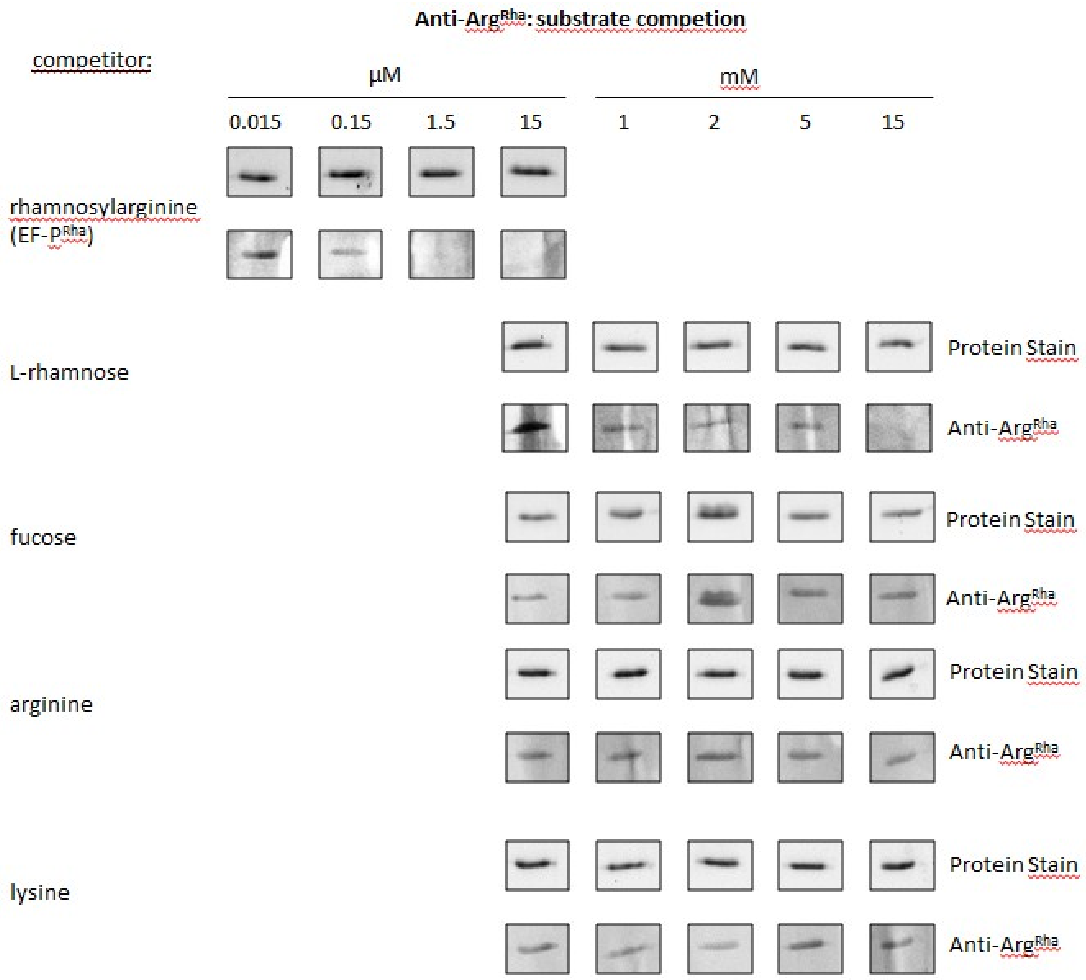

Fig S7 Detection of purified EF-P with Anti-Arg^{Rha}. Cross-reactivity analysis of Anti-Arg^{Rha} against L-rhamnose, L-fucose, L-arginine or L-lysine. 0.5 µg of purified EF-P^{Rha} were subjected to SDS-PAGE and subsequent Western Blot analysis using 0.2 µg/ml Anti-Arg^{Rha}. Anti-Arg^{Rha} was incubated with varying concentrations of EF-P^{Rha}, L-rhamnose, L-fucose, L-arginine and L-lysine. Buffer only served as control.

6. NMR spectrum

^{1}H-NMR of compound 1 (DMSO-d_6)

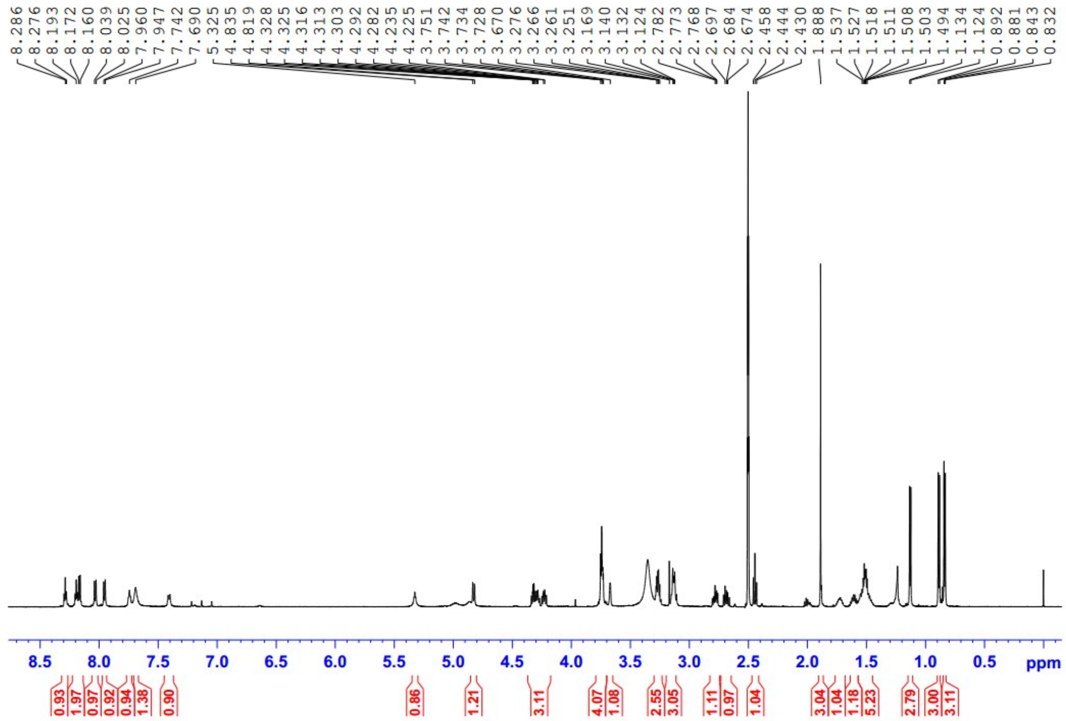

^{13}C-NMR of compound 1 (DMSO-d_6)

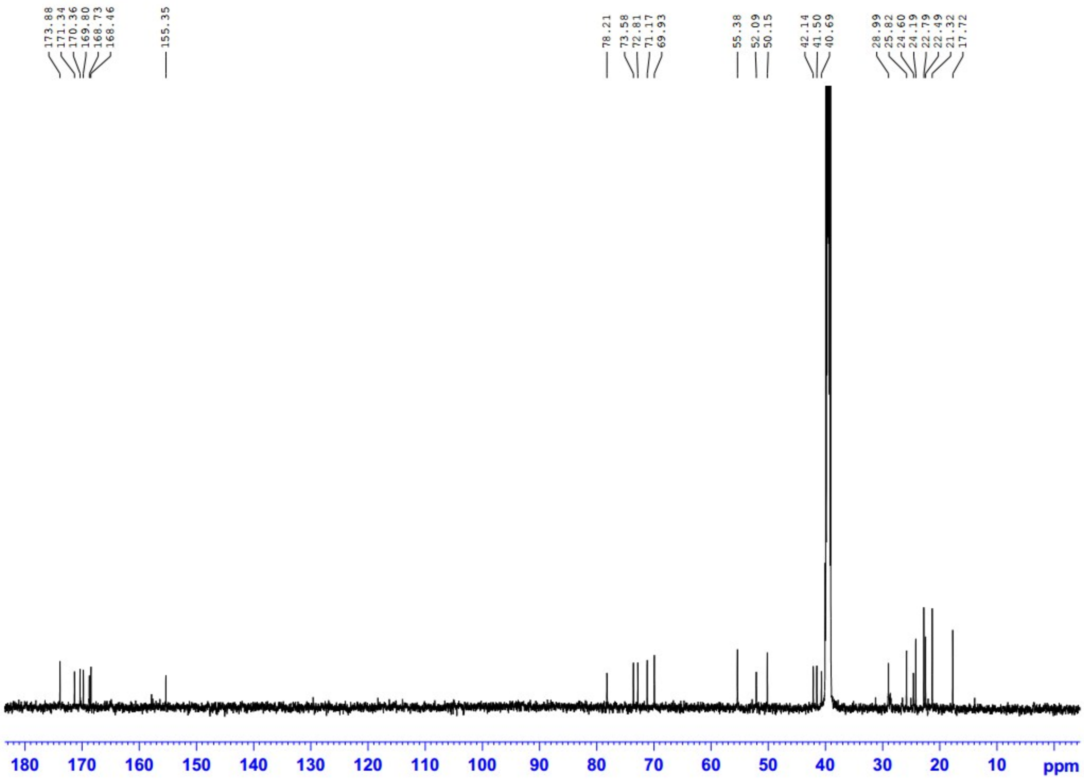

HR-Q-TOF-MS of compound 1

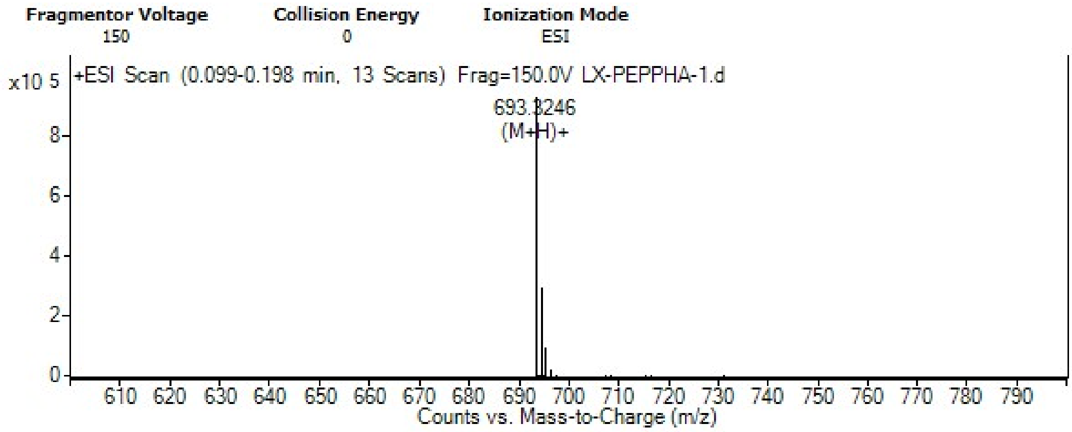

^{1}H-NMR of compound 3 ($CDCl_3$)

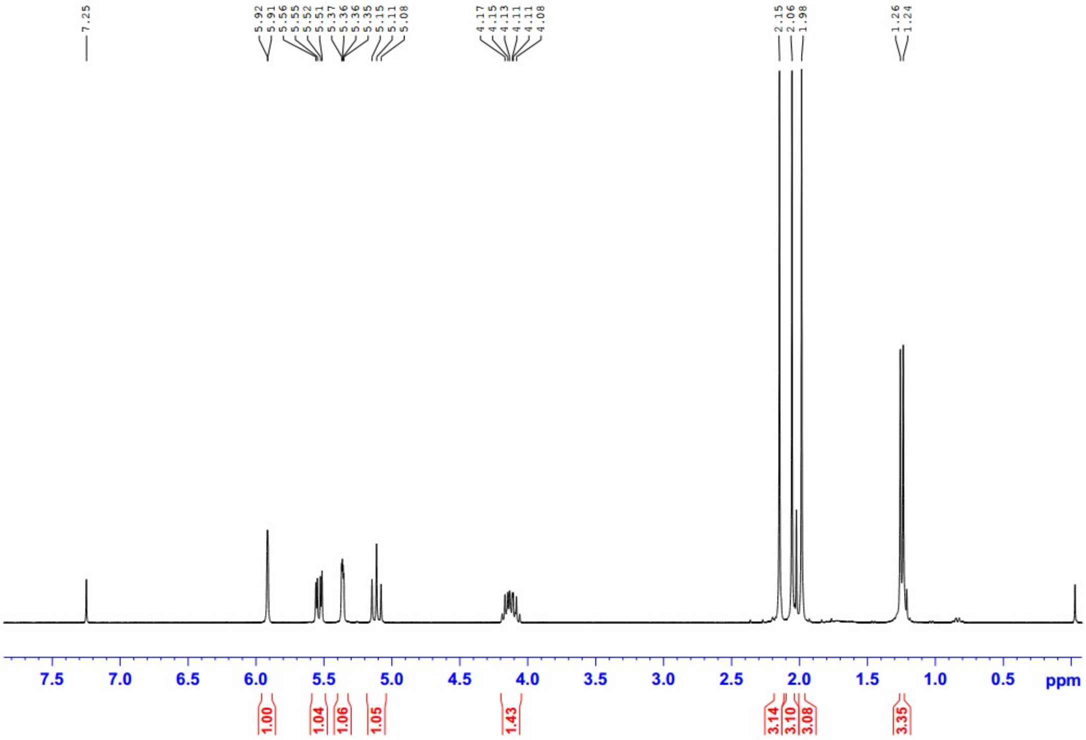

^{13}C-NMR of compound 3 ($CDCl_3$)

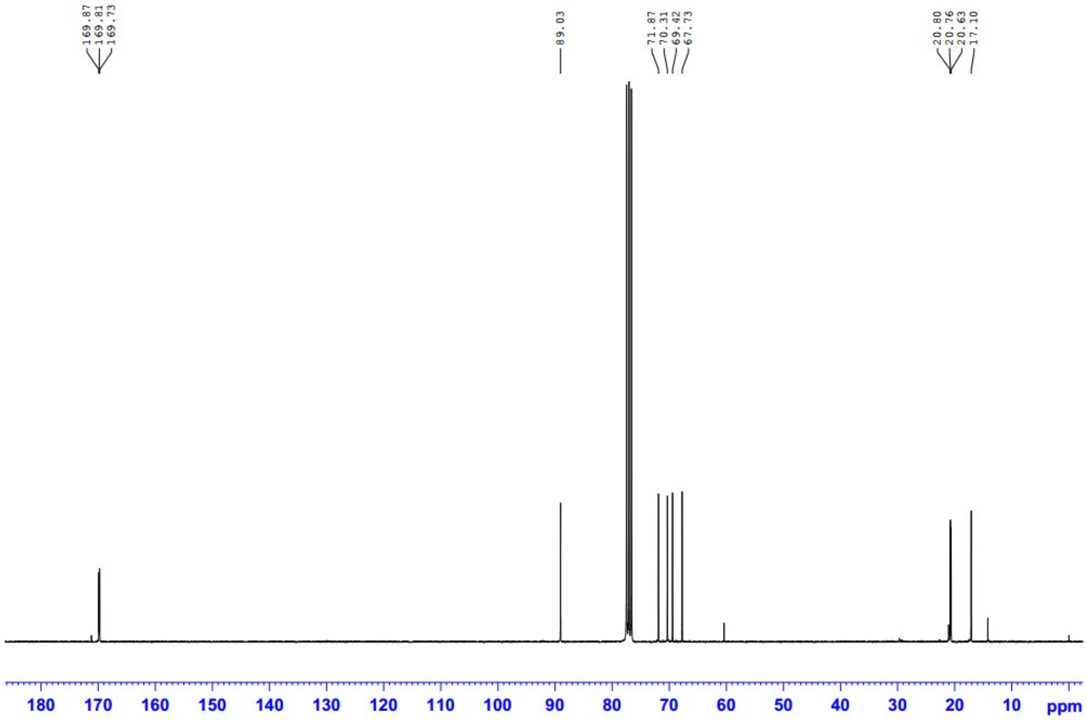

^{1}H-NMR of compound 4 ($CDCl_3$)

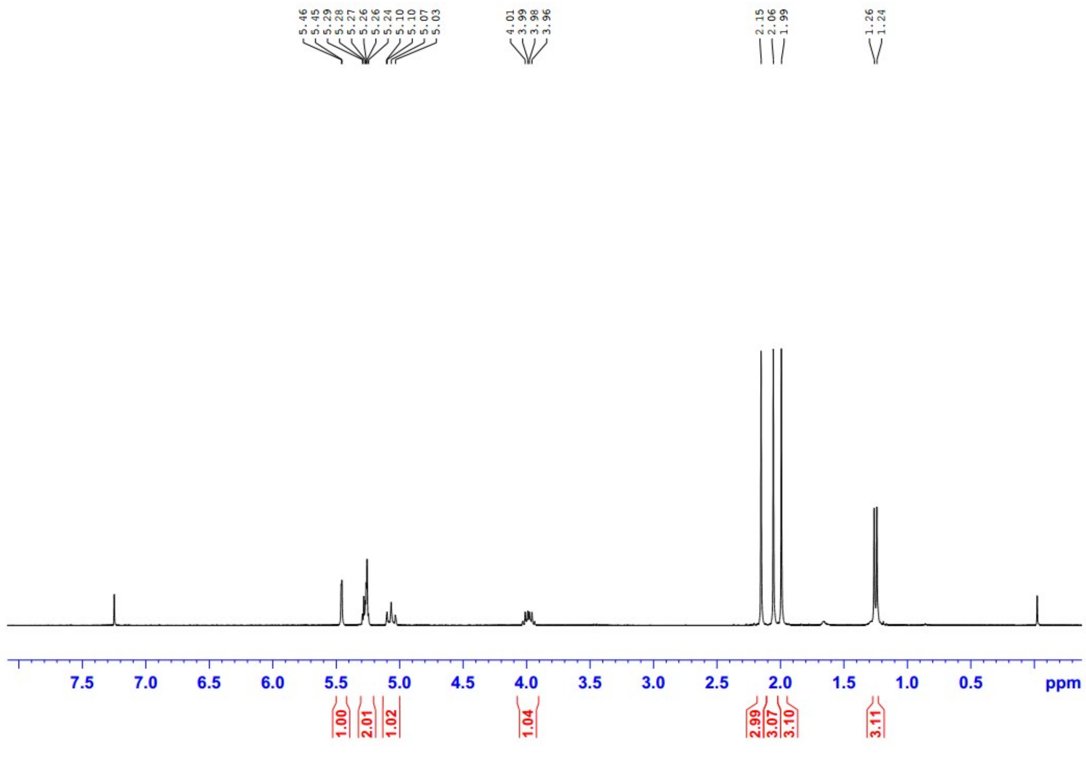

^{13}C-NMR of compound 4 ($CDCl_3$)

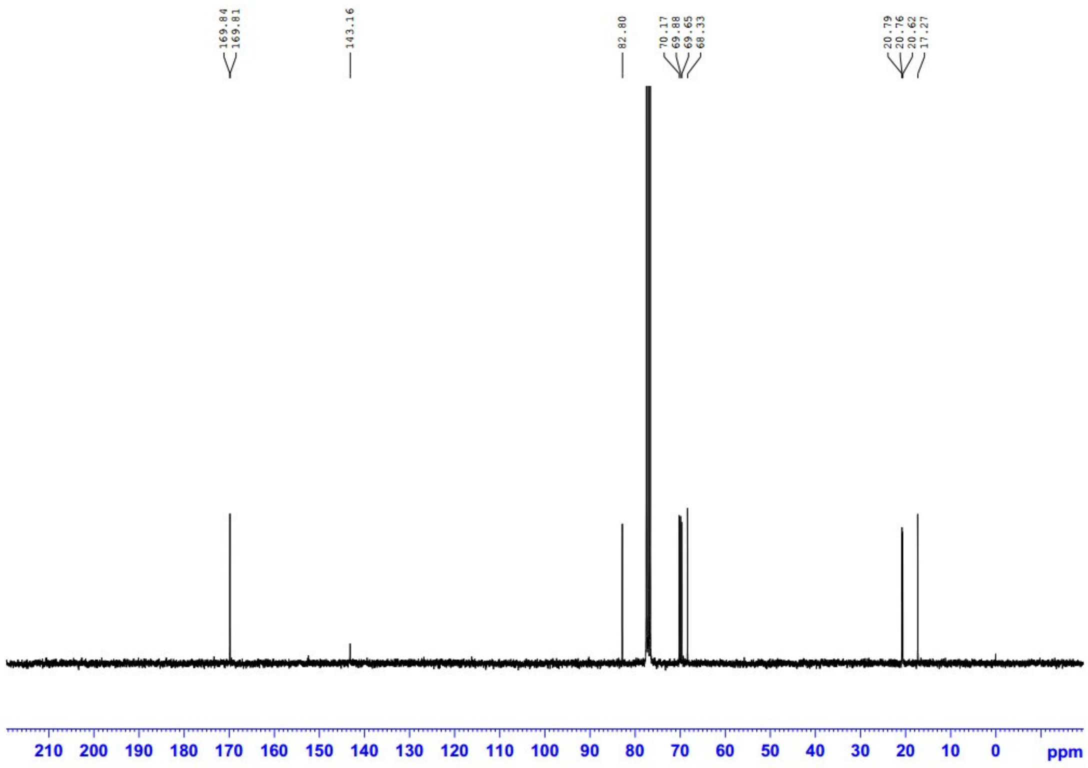

HR-Q-TOF-MS of compound 4

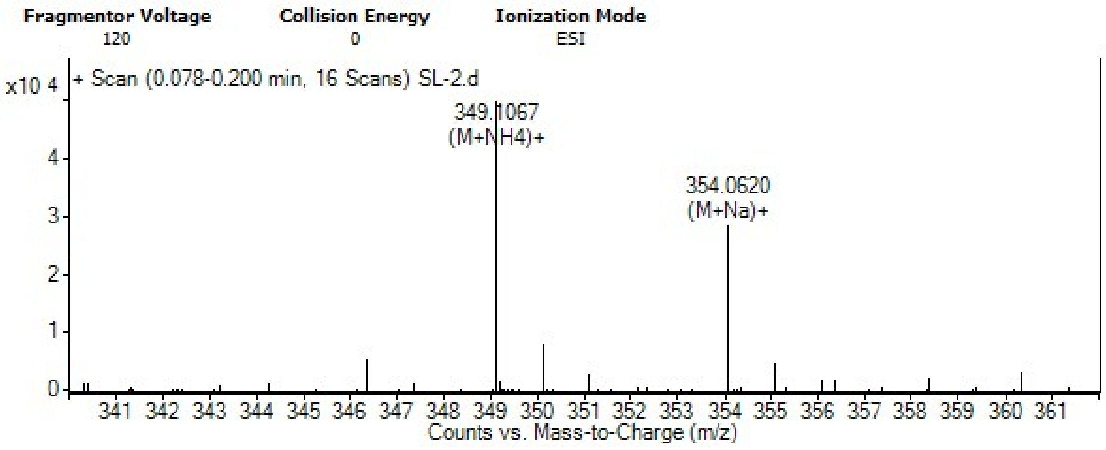

^{1}H-NMR of compound 5 ($CDCl_3$)

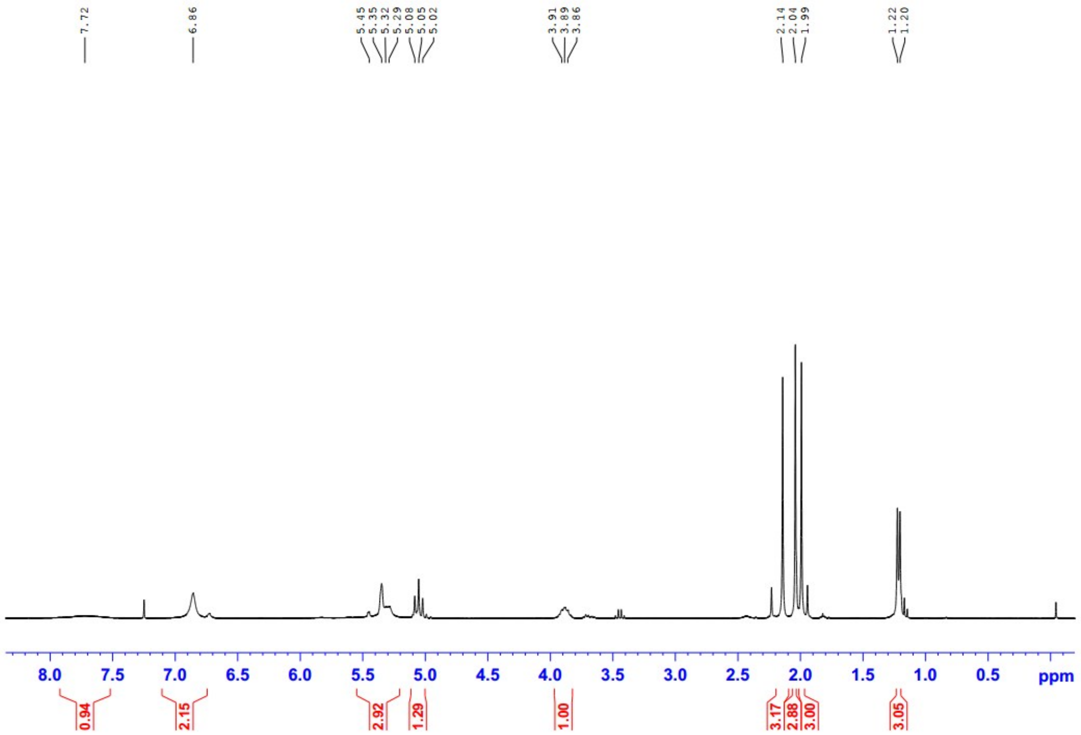

^{13}C-NMR of compound 5 ($CDCl_3$)

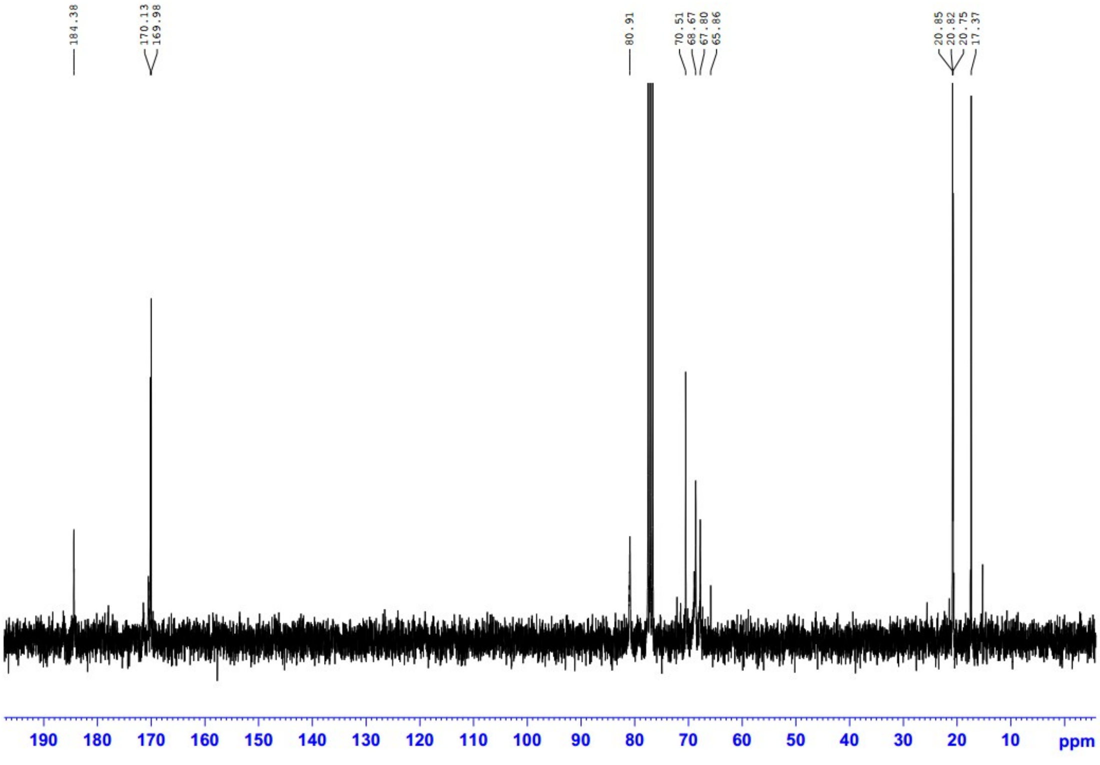

HR-Q-TOF-MS of compound 5

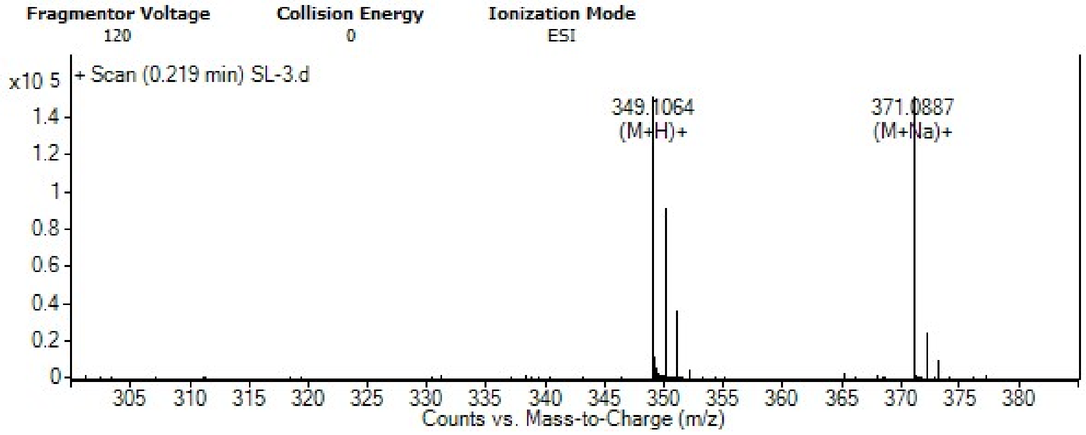

^{1}H-NMR of compound 6 ($CDCl_3$)

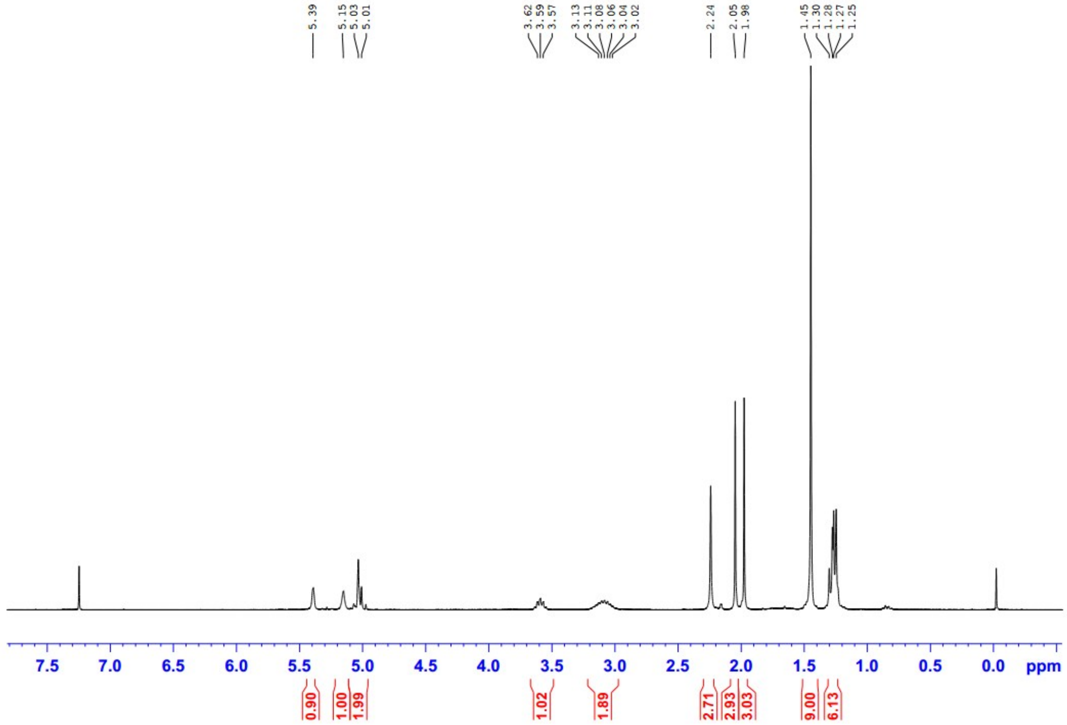

^{13}C-NMR of compound 6 ($CDCl_3$)

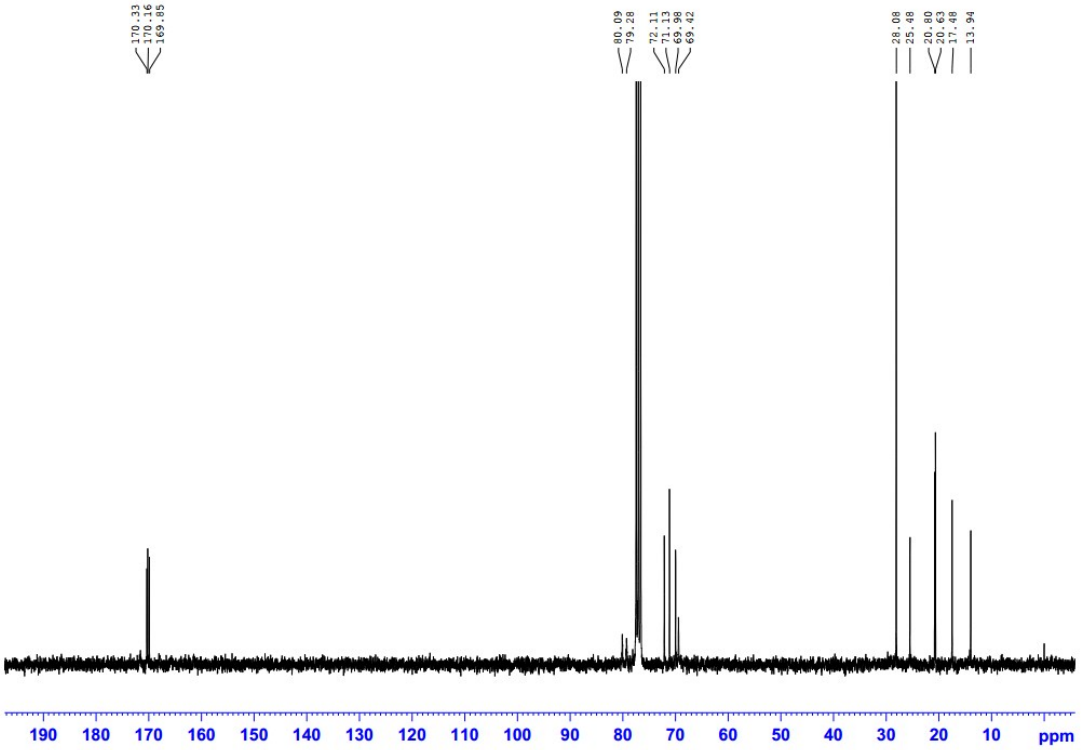

HR-Q-TOF-MS of compound 6

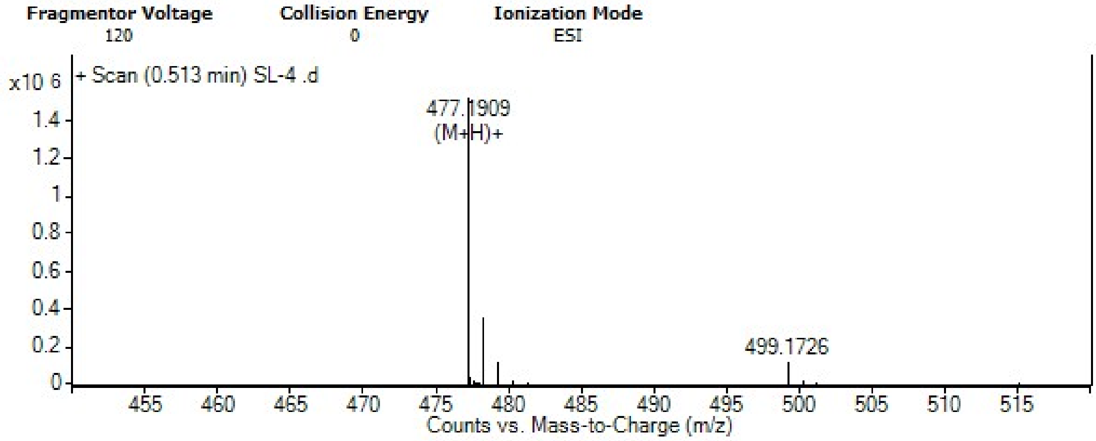

Table S2 Crystal data and structure refinement for compound **5**.

Identification code	a51230b	
Empirical formula	$C_{13}H_{20}N_2O_7S$	
Formula weight	348.37	
Temperature	298(2) K	
Wavelength	0.71073 Å	
Crystal system	Orthorhombic	
Space group	P 21 21 21	
Unit cell dimensions	a = 8.984(5) Å	α = 90°.
	b = 10.648(6) Å	β = 90°.
	c = 18.894(10) Å	γ = 90°.
Volume	1807.5(17) $Å^3$	
Z	4	
Density (calculated)	1.280 Mg/m^3	
Absorption coefficient	0.213 mm^{-1}	
F (000)	736	
Crystal size	0.660 x 0.200 x 0.150 mm	
Theta range for data collection	2.156 to 26.979 °	
Index ranges	-10<=h<=11, -13<=k<=13, -18<=l<=23	
Reflections collected	8825	
Independent reflections	3845 [R (int) = 0.0627]	
Completeness to theta = 25.242°	99.7 %	
Absorption correction	Semi-empirical from equivalents	
Max. and min. transmission	1.000 and - 0.009	
Refinement method	Full-matrix least-squares on F^2	
Data / restraints / parameters	3845 / 8 / 224	
Goodness-of-fit on F^2	0.841	
Final R indices [I>2 sigma(I)]	R1 = 0.0410, wR2 = 0.0809	
R indices (all data)	R1 = 0.0624, wR2 = 0.0860	
Absolute structure parameter	0.03(6)	
Extinction coefficient	n/a	
Largest diff. peak and hole	0.146 and -0.193 e.$Å^{-3}$	

7. References

[1] M. Sattler, J. Schleucher, C. Griesinger, *Prog. Nucl. Magn. Reson. Spectrosc.* **1999**, 34, 93-158.

[2] M. Sattler, M. Maurer, J. Schleucher, C. Griesinger, *J. Biomol. NMR* **1995**, *5*, 97-102.

[3] L. Kay, P. Keifer, T. Saarinen, *J. Am. Chem. Soc.* **1992**, *114*, 10663-10665.

[4] F. Schilling, L. R. Warner, N. I. Gershenzon, T. E. Skinner, M. Sattler, S. J. Glaser, *Angew. Chem.* **2014**, *126,* 4564–4568; *Angew. Chem.Int. Ed.* **2014**, *53*, 4475-4479.

[5] F. Delaglio, S. Grzesiek, G. W. Vuister, G. Zhu, J. Pfeifer, A. Bax, *J. Biomol. NMR* **1995**, *6*, 277-293.

[6] G. M. Sheldrick, *Acta. Crystallogr. A.* **2008**, *64*, 112-122.

[7] H. Inoue, H. Nojima, H. Okayama, *Gene* **1990**, *96*, 23-28.

[8] A. L. Starosta, J. Lassak, L. Peil, G. C. Atkinson, C. J. Woolstenhulme, K. Virumae, A. Buskirk, T. Tenson, J. Remme, K. Jung, D. N. Wilson, *Cell Rep.* **2014**, *9*, 476-483.

[9] M. M. Bradford, *Anal. Biochem.* **1976**, *72*, 248-254.

[10] U. K. Laemmli, *Nature* **1970**, *227*, 680-685.

[11] C. L. Ladner, J. Yang, R. J. Turner, R. A. Edwards, *Anal. Biochem.* **2004**, *326*, 13-20.

[12] J. Lassak, A. L. Henche, L. Binnenkade, K. M. Thormann, *Appl. Environ. Microbiol.* **2010**, *76*, 3263-3271.

[13] J. Lassak, E. C. Keilhauer, M. Furst, K. Wuichet, J. Godeke, A. L. Starosta, J. M. Chen, L. Sogaard-Andersen, J. Rohr, D. N. Wilson, S. Haussler, M. Mann, K. Jung, *Nat. Chem. Biol.* **2015**, *11*, 266-270.

[14] D. R. Macinga, M. M. Parojcic, P. N. Rather, *J. Bacteriol.* **1995**, *177*, 3407-3413.

[15] F. W. Studier, B. A. Moffatt, *J. Mol. Biol.* **1986**, *189*, 113-130.

[16] K. Venkateswaran, D. P. Moser, M. E. Dollhopf, D. P. Lies, D. A. Saffarini, B. J. MacGregor, D. B. Ringelberg, D. C. White, M. Nishijima, H. Sano, J. Burghardt, E. Stackebrandt, K. H. Nealson, *Int. J. Syst. Evol. Microbiol.* **1999**, *49 Pt 2*, 705-724.
